# Genome-wide identification and characterization of circular RNA m^6^A modification in pancreatic cancer

**DOI:** 10.1186/s13073-021-01002-w

**Published:** 2021-11-19

**Authors:** Ying Ye, Weiyi Feng, Jialiang Zhang, Kaiyu Zhu, Xudong Huang, Ling Pan, Jiachun Su, Yanfen Zheng, Rui Li, Shuang Deng, Ruihong Bai, Lisha Zhuang, Lusheng Wei, Junge Deng, Mei Li, Rufu Chen, Dongxin Lin, Zhixiang Zuo, Jian Zheng

**Affiliations:** 1 Sun Yat-sen University Cancer Center, State Key Laboratory of Oncology in South China and Collaborative Innovation Center for Cancer Medicine, Guangzhou, China; 2 Department of Pancreaticobiliary Surgery, Sun Yat-sen Memorial Hospital, Sun Yat-sen University, Guangzhou, China; 3 Department of Pathology, Sun Yat-sen University Cancer Center, Guangzhou, China; 4 Guangdong Provincial People’s Hospital & Guangdong Academy of Medical Sciences, Guangzhou, China; 5 Department of Etiology and Carcinogenesis, National Cancer Center/National Clinical Research Center/Cancer Hospital, Chinese Academy of Medical Sciences and Peking Union Medical College, Beijing, China; 6 Jiangsu Key Lab of Cancer Biomarkers, Prevention and Treatment, Collaborative Innovation Center for Cancer Medicine, Nanjing Medical University, Nanjing, China

**Keywords:** m^6^A modification, Circular RNA, m^6^A**-**seq, Pancreatic cancer

## Abstract

**Background:**

*N*^6^-methyladenosine (m^6^A) is the most abundant modification of RNA in eukaryotic cells and play critical roles in cancer. While most related studies focus on m^6^A modifications in linear RNA, transcriptome-wide profiling and exploration of m^6^A modification in circular RNAs in cancer is still lacking.

**Methods:**

For the detection of m^6^A modification in circRNAs, we developed a new bioinformatics tools called Circm6A and applied it to the m^6^A-seq data of 77 tissue samples from 58 individuals with pancreatic ductal adenocarcinoma (PDAC).

**Results:**

Circm6A performs better than the existing circRNA identification tools, which achieved highest F1 score among these tools in the detection of circRNAs with m^6^A modifications. By using Circm6A, we identified a total of 8807 m^6^A-circRNAs from our m^6^A-seq data. The m^6^A-circRNAs tend to be hypermethylated in PDAC tumor tissues compared with normal tissues. The hypermethylated m^6^A-circRNAs were associated with a significant gain of circRNA-mRNA coexpression network, leading to the dysregulation of many important cancer-related pathways. Moreover, we found the cues that hypermethylated m^6^A-circRNAs may promote the circularization and translation of circRNAs.

**Conclusions:**

These comprehensive findings further bridged the knowledge gaps between m^6^A modification and circRNAs fields by depicting the m^6^A-circRNAs genomic landscape of PDAC patients and revealed the emerging roles played by m^6^A-circRNAs in pancreatic cancer. Circm6A is available at https://github.com/canceromics/circm6a.

**Supplementary Information:**

The online version contains supplementary material available at 10.1186/s13073-021-01002-w.

## Background

m^6^A is the most abundant posttranscriptional modification in eukaryotic RNAs and play critical roles in various normal bioprocesses [1]. The m^6^A modification is deposited by the m^6^A methyltransferase complex (writer) composed of METTL3, METTL14, and WTAP and can be removed by m^6^A demethylases (erasers) such as FTO and ALKBH5. Distinct proteins (readers) can recognize m^6^A-modified mRNAs and decide their fates by affecting the stability, splicing, nuclear export, and translation of target RNAs [2, 3]. High-throughput m^6^A RNA immunoprecipitation sequencing (MeRIP-seq) studies revealed that m^6^A modification mainly occurs in messenger RNAs (mRNAs) with the consensus motif “RRm^6^ACH” (R = G or A; H = A, C or U) and is enriched near stop codons and in the 3′UTRs of mRNAs [4, 5].

Circular RNAs (circRNAs) are a special class of RNAs that are produced by a covalent linkage between the 5′ and 3′ ends of an RNA molecule [6]. In recent years, circRNAs have been intensively studied in a wide range of cells and tissues, revealing their critical roles in many important biological processes and diseases [7–9]. Compared to linear RNAs, circRNAs are highly stable and are frequently detected in exosomes and cell-free body fluids; therefore, circRNAs may be potential biomarkers or therapeutic targets in precise medicine [10, 11]. While most m^6^A studies focus on linear RNAs, a few studies show that m^6^A modifications also occur in circular RNAs [12], and m^6^A modifications affect the translation efficiency of circRNAs [13]. Moreover, there is evidence showing that m^6^A modifications in circRNAs play important roles in cancer progression [14]. However, to date, few studies on the transcriptome-wide mapping of m^6^A modifications in circRNAs in human cancer tissues have been reported.

Pancreatic ductal adenocarcinoma (PDAC) is an aggressive tumor that is typically diagnosed at an advanced stage and will become the second leading cause of cancer mortality within this decade [15, 16]. To date, there is still no efficient method for the treatment of PDAC, posing an urgent need for the development of new therapeutic strategies, which relies on a clearer understanding of cancer biology. Recent genome-wide association studies (GWAS) and whole-genome/exome/transcriptome sequencing studies on PDAC have revealed a complex network consisting of genetic and genomic/transcriptomic alterations that is closely associated with PDAC occurrence and development [17, 18]. There is widespread dysregulation of circRNAs, which has critical roles in cancer progression [9, 19]. However, little is known about the transcriptome mapping and functions of m^6^A modifications in circRNAs in PDAC.

In this study, we presented a de novo algorithm called “Circm6A” to detect m^6^A modification in circular RNAs from MeRIP-seq data, facilizing further researches on m^6^A modification in circRNAs. We then performed m^6^A sequencing (m^6^A-seq) on total RNAs of 53 tumor and 24 adjacent normal tissue samples from 58 individuals with PDAC and applied Circm6A to the m^6^A-seq data to explore the landscape of m^6^A modification in circRNAs. We found that approximately 23.1% of circRNAs in PDAC tissues harbored m^6^A modifications, and they tended to be hypermethylated in PDAC tumor tissues compared to adjacent normal tissues. Intriguingly, we found that hypermethylated m^6^As in circRNAs caused a gain of circRNA-mRNA coexpression in many cancer-related pathways. Moreover, our study also indicated that m^6^A modifications in circRNAs might promote the circularization and translation of circRNAs. Taken together, we provided a state-of-art tool to detect m^6^A modification in circRNAs, which will facilitate the following study of m^6^A modification in circRNA. Our findings shed new light on the function and mechanism of circRNA dysregulation in PDAC at RNA epigenetics level.

## Methods

### Construction of Circm6A

Circm6A (https://github.com/canceromics/circm6a) is a de novo algorithm for the detection of circRNAs and their m^6^A modifications from MeRIP-seq data. Circm6A will scan the sequencing alignment files from both MeRIP-seq IP (the IP library represents the RNA fragments captured by m^6^A-antibody pull-down) and INPUT (the paired INPUT library is derived from initial fragmented RNAs before immunoprecipitation) samples [4]. For the detection of circRNAs, Circm6A first detects junction reads with PCC signals (two segments of one read are aligned to the reference genome in a chiastic order) that reflect a circRNA candidate. Paired-end mapping signals (PEM signal, the paired mate of the BSJ read is properly aligned within the candidate circRNA region) are utilized for additional filtering when paired-end sequencing was used, as previously described [4], and this step will be automatically skipped in single-end sequencing data. Furthermore, Circm6A checks whether AG and GT dinucleotides and exon boundaries flank the back-splicing junction (BSJ) sites of candidate circRNAs. The identified circRNAs are annotated according to the GTF file downloaded from the GENCODE database (https://www.gencodegenes.org/). If only RNA-seq data is provided (parameter: -input input_sample.bam), Circm6A will only detect circRNAs from files.

For the detection of circRNAs with m^6^A modification (m^6^A-circRNAs), Circm6A will examine whether the circRNAs are significantly enriched in IP samples compared to INPUT samples in the MeRIP-seq data. Firstly, the whole genome is split into 25-bp bins. The read coverages in the genome bins around the BSJ sites of all identified circRNAs are calculated in the IP samples and INPUT samples, respectively. Then, the read coverage difference for the genome bins around BSJ sites between IP and INPUT samples are calculated using Fisher’s exact test. The *P* values from Fisher’s exact test are adjusted using the Benjamini-Hochberg method. A false discovery rate (FDR) of less than 0.05 is considered significant. The genome bins with significant enrichment in IP samples over INPUT samples are concatenated. If the concatenated bins are across the BSJ site, the circRNA with that BSJ site is considered as a candidate m^6^A-circRNA. Next, the loose and strict filters are applied to further filter the candidate m^6^A-circRNAs. If there is at least one BSJ read in IP samples (*N*_*BSJ*, *IP*_ ≥ 1), the circRNA was defined as low confidence m^6^A-circRNA. If the fraction of BSJ reads should be higher in IP samples than in INPUT samples ($\frac{N_{BSJ, IP}}{N_{total, IP}}:\frac{N_{BSJ, non- IP}}{N_{total, non- IP}}\ge 1.0\ \Big)$ and there is at least one BSJ read in IP samples (*N*_*BSJ*, *IP*_ ≥ 2), the circRNA was defined as high-confidence m^6^A-circRNA.

m^6^A peaks in linear RNAs are also detected according to previously published methods [4].

### Simulate MeRIP-seq data for performance evaluation

We developed an in-house simulator called MeRIP-simulator (https://github.com/canceromics/MeRIP-simulator) to simulate the MeRIP-seq data. GRCh38 genome FASTA and GTF annotation files (GENCODE version 25 [20], downloaded from https://www.gencodegenes.org/) were used as references for simulation.

For the simulation of MeRIP-seq Input sample, the linear genes were simulated based on the linear transcript information extracted from the GENCODE GTF annotation file and the circRNA genes were simulated based on the circRNA transcript information extracted from the five circRNA database (CircAtlas [21], MiOncoCirc [22], CSCD [23], TSCD [24], CIRCpedia [25]). A total of 18,689 circRNAs were simulated. The read counts of a transcript (*C*) were simulated using three coefficients: *C* = *F* · *d* · *l*, where *F* is the expression factor of the transcript, *d* is sequencing depth and *l* is the length of exons in the transcript. The expression factor of a transcript is defined as *F* = *e*^*λ*^ and *λ* is based on a Poisson distribution *P*(*λ* = 1.0). The sequencing depth is given by user. The read length *R* follow a binominal distribution $R\sim B\left(\left\lceil \frac{L_r}{0.95}\right\rceil, 0.95\right)$, where *L*_*r*_ means expected read length given by Tuser or 150 bp by default. We let the insert size *S* follow another binominal distribution *S*~*B*(*L*_*s*_ · 2, 0.5), where *L*_*s*_ means expected insert size given by user or 300 bp by default. The simulated reads will be outputted as FASTQ format.

For the simulation of MeRIP-seq IP sample, the m^6^A sites were simulated based on m^6^A peaks obtained from public MeRIP-seq dataset (GSE120229 [26]: SRR7881528 and SRR7881532). The m^6^A peak was called using STAR [27] and MACS2 [28]. The parameters of STAR were “--twopassMode Basic --chimSegmentMin 20 --outFilterIntronMotifs RemoveNoncanonical --outFilterMultimapNmax 20 --alignIntronMin 20 --algigIntronMax 1000000 --alignMatesGapMax 1000000”. The cutoff of the *P* value for significant peak for MACS2 was set at 1.0e−6. Two library construction strategies (fragmentation of RNA before MeRIP and fragmentation of RNA after MeRIP) were simulated. For the strategy of fragmentation of RNA after MeRIP, the read coverages of the linear and circular transcripts with m^6^A modifications were simulated 20-fold higher than the read coverages of those linear and circular transcripts without m^6^A modifications. For the strategy of fragmentation of RNA before MeRIP, the read coverages of the m^6^A regions (200 bp) in linear and circular transcripts were simulated 20-fold higher than the read coverages of non-m^6^A regions. We also simulated background reads to mimic the nonspecific IP of m^6^A antibody in IP sample and their abundance was 0.01-fold of that in INPUT sample.

### Collection of PDAC samples

A total of 77 (53 cancer and 24 normal) tissues were obtained from pancreatic cancer patients who underwent pancreatectomy at the Sun Yat-sen University Cancer Center and Sun Yat-sen Memorial Hospital between 2010 and 2018 (detailed in Additional file 1: Table S1). The PDAC tumor and non-tumor tissue (≥ 5 cm away from tumor) samples were collected at surgery from each patient and immediately placed in liquid nitrogen. All patients received no chemotherapy or radiotherapy before surgery. The diagnosis of PDAC was histopathologically confirmed and tumor stage was classified according to the 7th edition of AJCC Cancer Staging System [29]. Tumor samples and distant normal tissues were embedded in optimal cutting temperature medium, and histological sections stained with hematoxylin and eosin were reviewed by at least two pathologists for quality assurance that tumor specimens contained at least 60% tumor cell nuclei, whereas normal specimens contained no tumor cells. Clinical data for PDAC patients were collected, which included age at diagnosis, differentiation, lymph node metastasis, vascular invasion, neural invasion, tumor stage, and treatment. The survival time of individuals with PDAC was measured from the date of diagnosis to the date of last follow-up or death. Whether and when a subject had died were obtained from inpatient and outpatient records, subjects’ families, or through follow-up telephone calls.

### Public RNA-seq data

We included two separate independent circRNA-related studies [7, 30], which included control (only rRNA-depleted) samples and matched samples additionally treated with RNase R. The first dataset contains 4 runs of RiboMinus RNA-Seq libraries of the HeLa cell line downloaded from the NCBI Sequence Reads Archive (accession numbers: SRR1636985, SRR1636986, SRR1637089, and SRR1637090) [31]. The second public dataset comprises 2 runs of rRNA-depleted RNA-seq data derived from the Hs68 cell line (accession numbers: SRR445016 and SRR444975) [7].

### Public CLIP-seq data

All reported binding sites for “readers” (YTHDF1-3, YTHDC1-2, IGF2BP1-3, HNRNPC, and HNRNPA2B1) and EIFs (EIF3A, EIF3B, EIF3D, EIF3G, EIF3H, EIF4A3, EIF4G2) were obtained from POSTAR2 [32]. MeRIP-seq data of hESC cell line were retrieved from Gene Expression Omnibus (GEO accession number: GSE55572 [33] and GSE85324 [12]). ADAR binding sites of hESC cell line were obtained from a public iCLIP-seq study (GEO accession number: GSE63709 [34]).

### Detection of circRNAs in public and simulated data using eight known tools

For each public and simulated RNA-seq sample, circRNA detection was also performed using other eight known tools (ACFS [35], AutoCirc [12], circRNA_finder [36], CIRI2 [31, 37], CIRCexplorer2 [38], DCC [39], Find Circ [8], and MapSplice [40]) according to the suggested parameters of each tool. RNase R-resistant (enriched) circRNAs were defined as circRNAs with at least a 5-fold enrichment of abundance in RNase R-treated samples, RNase R-sensitive (depleted) circRNAs were defined as those circRNAs with an abundance reduction in RNase R-treated samples, and RNase R-unaffected circRNAs were defined as those with a 1~5-fold change in RNase R-treated samples, as previously described [41].

### Sample processing

Total RNA from tissue samples was extracted with TRIzol reagent (Invitrogen). RNA samples were quantified by measuring the absorption value at 260 nm with a UV spectrophotometer and then analyzed via the RNA6000 Nano assay (Agilent) for determination of an RNA integrity number (RIN), and only analytes with an RIN ≥ 7.0 were included in this study.

### m^6^A-specific methylated RNA immunoprecipitation and high-throughput sequencing (MeRIP-seq)

Total RNA from tissue samples was isolated using TRIzol (Life Technologies), digested by DNase I (NEB), and then subjected to two rounds of RiboMinus treatment to reduce rRNA content (Illumina). For m^6^A immunoprecipitation, the Magna MeRIP m^6^A Kit (Millipore) was used according to the manufacturer’s instructions. Briefly, 20 μg of Ribo-off-treated RNA was sheared to approximately 100–200 nt in length by metal-ion-induced fragmentation, purified, and incubated with 10 μg of anti-m^6^A antibody (Synaptic Systems, 202003)-conjugated beads in 500 μL 1× immunoprecipitation buffer supplemented with RNase inhibitors at 4 °C overnight. The m^6^A-modified RNA was recovered by treatment with proteinase K, acidic phenol/chloroform extraction, and ethanol precipitation. Sequencing libraries were prepared using the Illumina protocol, and sequencing was performed on an Illumina HiSeq2500.

### m^6^A peak-specific qRT-PCR

Total RNA from tissue samples was isolated according to the procedure shown above. For RNase R treatment, 20 μg of total RNA was incubated for 15 min at 37 °C with or without 5 U/μg RNase R (Epicenter Technologies). For MeRIP-qPCR, the RNA samples were fragmented and immunoprecipitated by anti-m^6^A antibody as described above. The purified m^6^A-containing RNAs were reverse transcribed using a RevertAid First Strand cDNA Synthesis Kit (Thermo) with random hexamers. The enrichment of m^6^A was quantified via quantitative real-time PCR in triplicate on a Roche LightCycler 480 using the SYBR Green method. Gene-specific primers are shown in Additional file 1: Table S2.

### Quantitative real-time PCR (qRT-PCR) analysis

Total RNA from tissue samples was isolated and subjected to RNase R treatment according to the procedure shown above. Reverse transcription for mRNA and circRNAs was performed with a RevertAid First Strand cDNA Synthesis Kit (Thermo) using random hexamers. Relative RNA levels were determined by qRT-PCR in triplicate on a Roche LightCycler 480 using the SYBR Green method. β-Actin was employed as an internal control for quantification of the level of each gene. The primer sequences for the analyzed genes are summarized in Additional file 1: Table S2. The relative expression levels were calculated according to the 2^−ΔCT^ method.

### Cell lines and cell culture

Human PDAC cell line PANC-1 was purchased from the Cell Bank of Type Culture Collection of the Chinese Academy of Sciences Shanghai Institute of Biochemistry and Cell Biology. PANC-1 was maintained in DMEM medium supplemented with 10% fetal bovine serum in an atmosphere of 5% CO_2_ and 99% relative humidity at 37 °C. Cells passaged for < 6 months were authenticated by DNA fingerprinting analysis using short-tandem repeat (STR) markers.

### RNA interference

PDAC cells were pre-seeded in 6 cm dish with 50–60% confluency before night. Small interfering RNA (siRNA) targeting METTL3 and scramble control ((Additional file 1: Table S2) were purchased from GenePharma. Transfection of siRNA (75 nM) or scramble control was performed using lipofectamine 3000 (Life Technologies) according to the manufacturer’s instructions. The transfected cells were harvested 48 or 72 h after transfection and followed by downstream application. Efficiency of RNA interfering was validated by qRT-PCR as described above.

### Metabolic labeling of nascent RNAs with 4sU and nascent RNA purification

Metabolic labeling of newly transcribed RNAs was performed as described previously [42, 43]. PANC-1 cells were incubated with 100 μM 5, 6-dichloro-1-β-D-ribofuranosylbenzimidazole (DRB) for 3 h to block Pol II transcription. Transcription recovered after DRB release and newly transcribed RNAs were labeled with 200 μM 4sU. After terminating transcription with TRIzol treatment, 200 μg total RNAs were used for biotinylation and purification of 4sU-labeled nascent RNA. After incubating with 0.2 mg/mL EZ-link biotin-HPDP (Pierce, 21341, dissolved in dimethylformamide (DMF, Sigma, D4551) at a concentration of 1 mg/mL) in biotinylation buffer (10 mM Tris PH 7.4, 1 mM EDTA) at room temperature (RT) for 1.5 h with rotation, bound biotin-HPDP were purified with chloroform and precipitated using equal volume of isopropanol and 1:10 volume of 5 M NaCl. Biotinylated 4sU-labeled RNAs were isolated using 150 μL streptavidin-coated magnetic beads (Invitrogen) for 30 min at RT. After substantially washed on beads, nascent RNAs were eluted twice with 100 μL 0.1 M dithiotheitol (DTT) and precipitated with 40 μL of 4 M LiCl, 2 μL glycogen, and 600 μL ice-cold ethanol.

### RNA immunoprecipitation assays

RNA immunoprecipitation (RIP) assays were performed using the Magna RIP RNA-Binding Protein Immunoprecipitation kit (Millipore). 2 × 10^6^ Cells were rinsed twice with ice-cold phosphate buffered saline (PBS), harvested and then centrifuged at 1000 rpm for 5 min at 4 °C. Cell lysates were centrifuged at 12,000 rpm for 15 min at 4 °C and the supernatants were precleared with 15 μL Dynabeads Protein G (Invitrogen) to get rid of nonspecific binding. Then, the precleared lysates were used for IP by incubating with anti-EIF3A (ab86146) or anti-EIF3B (ab133601) antibodies from Abcam or anti-EIF3H (#PA5-87290) antibody from Invitrogen at 4 °C overnight with rotation. Protein-RNA mixture were substantially washed on beads, followed by extraction with phenol/chloroform/isoamyl alcohol and subjected to qRT-PCR using the primers listed in Additional file 1: Table S2.

### Polysome profile analysis

PANC-1 cells (1 × 10^7^) were treated with 100 mg/mL cycloheximide (Sigma-Aldrich) at 37 °C for 5 min, washed twice with ice-cold PBS containing 100 mg/mL cycloheximide, and lysed with 500 μL polysome lysis buffer (15 mM Tris-HCl, 5 mM MgCl_2_, 100 mM KCl, 2 mM DTT, 1% Triton X-100, 100 μg/mL cycloheximide, 1 mg/mL heparin sodium). Cell lysates were incubated on ice for 10 min and centrifuged at 16,000×*g* at 4 °C for 7 min. The supernatant was loaded onto top of a 5–50% sucrose gradient followed by centrifuging at 222,228×*g* at 4 °C for 120 min (Beckman). The gradient was divided and collected into 15 fractions by monitoring RNA absorbance at 254 nm with an ISCO fractionator (Brandel). RNA from each fraction was extracted using TRIzol reagent (Invitrogen) and analyzed by qRT-PCR. RNA distributions across the polysome profile are presented as percentages.

### Identification and quantification of circRNAs in PDAC and normal tissue samples

Reads were first mapped to the human reference genome (GRCh38) by BWA (v0.7.15) [44] and the annotation file obtained from the GENCODE database (https://www.gencodegenes.org/). Circm6A was applied to detect the circRNAs and their m^6^A modifications from all our PDAC tissue and normal tissue samples. Only circRNAs that had read counts ≥ 2 and were detected in at least 2 samples were retained. The circRNA expression level was calculated by the spliced reads per billion mapped reads (SRPBM) method, as previously mentioned [7]. Differential circRNA abundance analysis was carried out with edgeR [45] based on the quantification results from Circm6A. The thresholds for differential expression were a *P* ≤ 0.05 and a fold change ≥ 1.5 between PDAC and normal tissue samples.

### Construction of a random forest model for prediction of m^6^A modifications in circRNA

m^6^A-circRNAs identified by Circm6A were defined as the positive set. Non-m^6^A-circRNAs identified by Circm6A were defined as the negative set. We included some significant differential features of circRNA characteristics, the relative position between a circRNA and the m^6^A peak region of colinear transcripts, and related features of host genes as previously reported [46]. Finally, the top 14 features were selected for the construction of the random forest model (detailed in Additional file 1: Table S3).

### Relative m^6^A level quantification and differential methylation analysis

The relative m^6^A level for each peak of m^6^A-circRNA was quantified according to the approach described by Schwartz et al. [33]. Briefly, the read coverage for each peak in IP and INPUT (control) samples was calculated during the peak-calling module of Circm6A. The spliced reads per billion mapped reads (SRPBM) method was then used to normalize the read coverage. The relative m^6^A level was obtained by calculating the ratio between the IP SRPBM value and the INPUT SRPBM value for each m^6^A-circRNA. To obtain the dysregulated m^6^A modifications of circRNAs between PDAC and normal tissue samples (DM circRNAs), we utilized the Wilcoxon rank sum test on common peaks between PDAC and normal tissue samples (FDR ≤ 0.05; absolute fold change ≥ 1.5). CircRNAs that were specifically m^6^A modified in PDAC samples were also included in the hypermethylated-m^6^A-circRNAs; in the same way, those specifically m^6^A modified in normal samples were included in the hypo-m^6^A-circRNAs.

### Survival analysis with the Cox proportional hazards model

To independently investigate the prognostic role of each hyper-/hypomethylated m^6^A-circRNA, the relative m^6^A level of the dysregulated m^6^A-circRNAs, survival data in terms of OS/PFS and other clinical features, such as sex, age, were subjected to multivariate analysis using the Cox proportional hazards model. Hyper-/hypomethylated m^6^A-circRNAs with an FDR ≤ 0.25 were identified as significantly correlated with PFS/OS.

### mRNA quantification and differential expression analysis

mRNA levels were quantified using RSEM [47] (parameters: “-paired-end, -star”) on RiboMinus-treated (control/input) RNA-seq data. mRNA expression was measured in fragments per kilobase per million (FPKM). The “DESeq2” R package [48] was applied for differentially expressed mRNAs (DE mRNAs). mRNAs with an expression absolute fold change ≥ 1.5 and an FDR ≤ 0.05 were identified as DE mRNAs.

### Construction of the circRNA-mRNA coexpression network

Similarities between circRNA and mRNA expression patterns were determined by computing a Pearson correlation coefficient matrix for each circRNA-mRNA pair, and pairs with a correlation (*r)* ≥ 0.5 were defined as coexpression pairs. To avoid false positives in the coexpression network analysis, the nodes on the network were restricted to mRNAs or circRNAs expressed in at least 3 tissues, as previously described [21]. TargetScan miRNA site predictions were used to find RNAs (circRNAs and mRNAs) with MREs [49], and miRTarBase [50] was used as a further experimentally validated source. Coexpression pairs sharing the same MERs were ceRNAs [21].

### Pathway enrichment analysis

All pathway enrichment analyses of KEGG and GO terms were performed with clusterProfiler [51]. KEGG pathways or GO terms with an FDR ≤ 0.05 were considered significantly enriched.

### Permutation test

The Bioconductor package regioneR [52] was employed to test the significance of the overlap between two sets of regions. Peak sets were shuffled 10,000 times for each of the permutation tests. The widths of the peaks and total number of peaks from the tested sets were used for the widths of the random peaks. The following options were used for all three permutation tests: permTest (ntimes = 10,000, randomize.function = randomizeRegions, evaluate.function = numOverlaps, count.once = TRUE).

## Results

### De novo algorithm to identify m^6^A modification in circRNAs from m^6^A-seq data

**Fig. 1:**
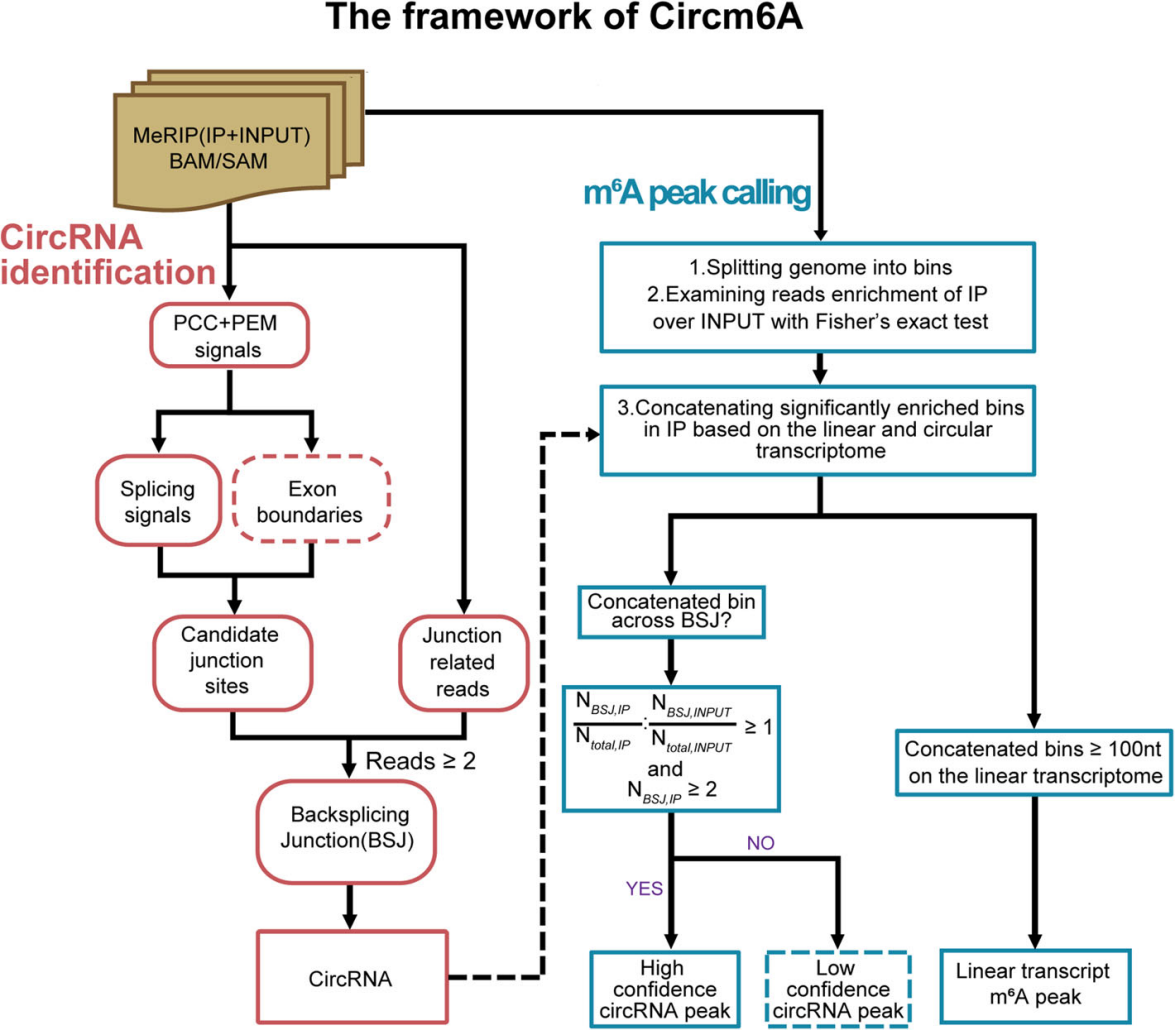
The framework of Circm6A. The pipeline of Circm6A consists of two steps: first, identification of circRNAs from MeRIP-seq IP and INPUT samples according to PCC and PEM signals; second, identification of circRNAs and linear transcripts with m^6^A modifications by examining whether the circRNAs or regions in linear transcripts are significantly enriched in IP samples compared to INPUT samples in the MeRIP-seq data. PCC, paired chiastic clipping signals; PEM, paired-end mapping signals

We developed a de novo algorithm called Circm6A to detect circRNAs and their m^6^A modifications from m^6^A-seq data. The workflow of Circm6A is illustrated in Fig. 1. First, Circm6A identifies circRNAs by searching back-splicing junction (BSJ) reads with paired chiastic clipping signals (PCC signals) from both MeRIP-seq IP and INPUT read alignments. If paired-end data are dealt with, paired-end mapping signals (PEM signals) will be automatically utilized to check that mapping of mates properly matches within the relevant circRNAs, as previously described [31]. To further remove potential false positives, the candidate circRNAs are filtered with additional features such as splicing signals (such as “GT-AG”) and exon boundaries. Next, we identify the circRNAs with m^6^A modification (m^6^A-circRNAs) by examining whether the circRNAs are significantly enriched in IP samples compared to INPUT samples. To do this, we count the reads coverage in the up- and downstream 100-bp regions around the BSJ sites of all circRNAs in the IP samples and INPUT samples, respectively. We split the 200-bp BSJ region into 25-bp bins and concatenate the bins with significant enrichment in IP samples over INPUT samples (Fisher’s exact test). If the concatenated bins are across the BSJ site and there is at least one BSJ read in IP samples, the circRNA with that BSJ site is considered as a m^6^A-circRNA. To obtain high-confidence m^6^A-circRNAs, we apply additional filters: first, there should be more than one BSJ reads in IP samples; second, the fraction of BSJ reads should be higher in IP samples than in INPUT samples. In addition, m^6^A peaks in linear RNAs are also detected according to previously published methods [4]. The full method and implementation details are given in the “Methods” section.

**Fig. 2:**
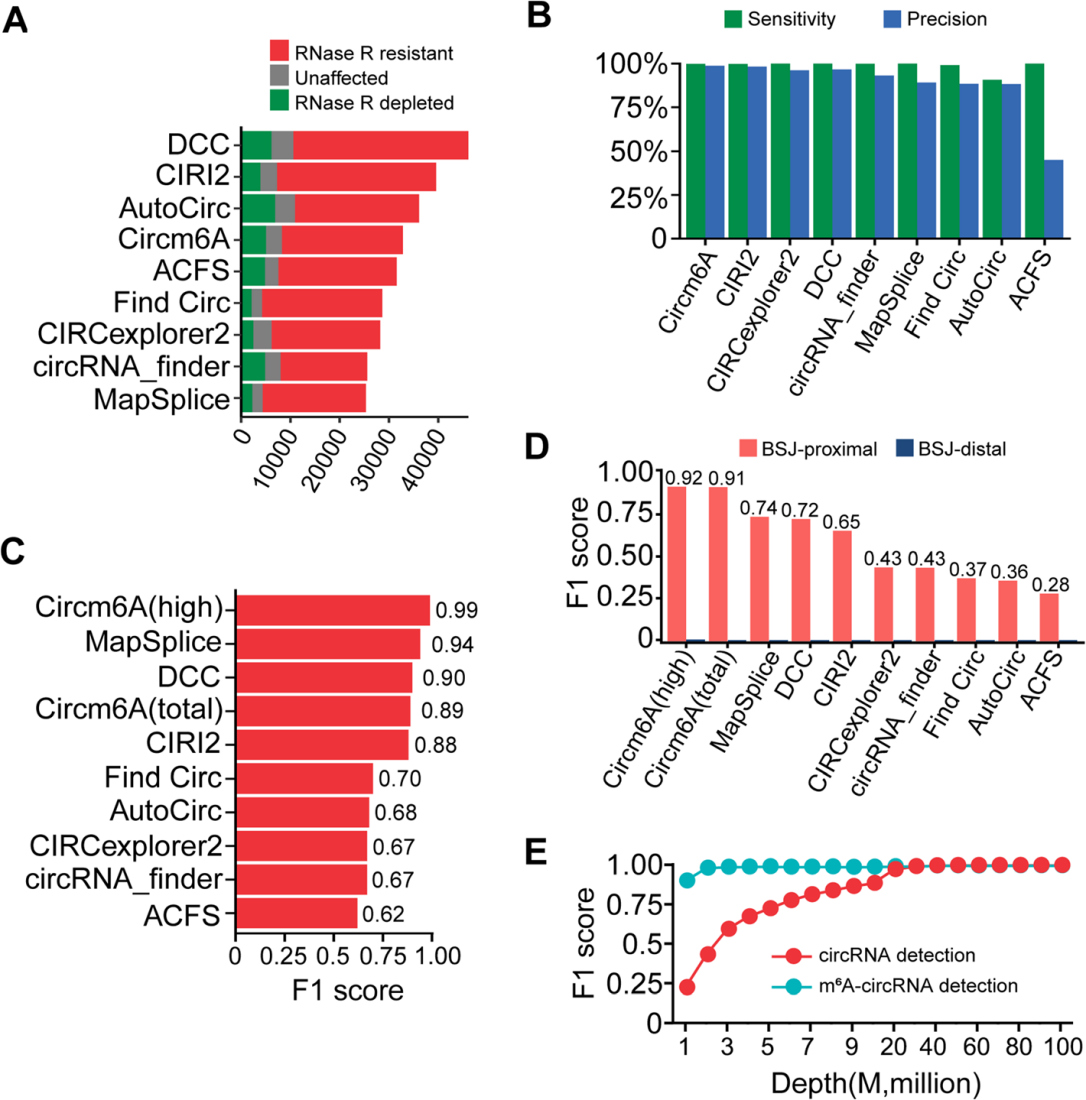
Performance evaluation of Circm6A. **A** Bar plot showing the performance of Circm6A and other eight known circRNA detection tools in the identification of circRNAs using public RNA-seq dataset consist of RNase R-treated or untreated samples. RNase R-depleted represents circRNAs that were depleted after RNase R treatment. Unaffected represents circRNAs that were enriched 1–5-fold after RNase R treatment. RNase R resistant represents circRNAs that were enriched ≥ 5-fold after RNase R treatment. **B** Bar plot showing the performance of Circm6A and other eight known circRNA detection tools in the identification of circRNAs using simulated datasets. **C** Bar plot showing the performance of Circm6A and other eight known circRNA detection tools in the identification of m^6^A-circRNAs using simulated MeRIP-seq datasets with fragmentation of RNA after MeRIP. **D** Bar plot showing the performance of Circm6A and other eight known circRNA detection tools in the identification of m^6^A-circRNAs using simulated MeRIP-seq datasets with fragmentation of RNA before MeRIP. BSJ-proximal, distance of m^6^A in circRNAs to BSJ within fragment length; BSJ-distal, m^6^As of circRNAs distal to BSJ (distance to BSJ over fragment length). **E** Saturation curve to access the impact of sequencing depth on the identification of circRNAs and m^6^A-circRNAs, respectively. M, million

To evaluate the performance of Circm6A in the identification of circRNAs, we compared Circm6A with eight known circRNA detection tools including ACFS [35], AutoCirc [12], circRNA_finder [36], CIRI2 [31, 37], CIRCexplorer2 [38], DCC [39], Find Circ [8], and MapSplice [40], which apply different strategies to detect circRNAs. The fraction of RNase R-depleted circRNAs is usually used to represent the false positives [41]. By utilizing two public datasets (HeLa and Hs68 cell lines) that consist of RNase R-treated or untreated samples, we calculated the fraction of RNase R-depleted circRNAs by comparing the circRNA level in the RNase R-treated samples with that in untreated samples. As a result, Circm6A detected 32836 circRNAs in the two public datasets and 15.4% of circRNAs were RNase R-depleted, which was comparable to the performance of the other eight tools (Fig. 2A). Moreover, we evaluated the performance with simulated RNA-seq datasets generated with our in-house simulator. Among the simulated datasets, Circm6A achieved the highest F1 score (0.99) compared to other eight tools (average F1 score: 0.91, F1 score: 0.62 ~ 0.94) (Fig. 2B and Additional file 1: Table S4).

To further evaluate the performance of Circm6A in the identification of m^6^A-circRNAs, we developed an in-house simulator (MeRIP-simulator, see “Methods” section) to simulate MeRIP-seq data. There are two MeRIP-seq library construction strategies, one which conducts fragmentation of RNA before m^6^A antibody IP (MeRIP) and the other conducts fragmentation of RNA after MeRIP, which theoretically have great impact on the identification of m^6^A-circRNAs (Additional file 1: Fig. S1A). Therefore, we evaluated the performance of Circm6A by simulating both of the two MeRIP-seq library construction strategies. For the strategy of RNA fragmentation after MeRIP, we simulated three MeRIP-seq datasets with sequencing depth range from 60 to 80 million reads. Previous study on genome-wide detection of m^6^A-circRNAs treated those circRNAs detected in IP sample as m^6^A-circRNAs [12]. Due to the nonspecific binding of m^6^A antibody, we hypothesized that some circRNAs without m^6^A modification will be randomly pulled down in IP samples. Therefore, taking all the circRNAs detected in IP samples as m^6^A-circRNAs might cause false discoveries. To avoid false discoveries, Circm6A utilizes Fisher’s exact test to examine whether the circRNAs are enriched in IP sample compared to INPUT sample (Additional file 1: Fig. S1B). We compared Circm6A with eight known circRNA detection tools using the simulated MeRIP-seq datasets. As expected, Circm6A has highest F1 score (0.99) compared to the approach of detecting circRNAs in IP sample using the eight known circRNA detection tools (average F1 score 0.76, F1 score 0.62 ~ 0.94) (Fig. 2C and Additional file 1: Table S5). Even if low confidence m^6^A-circRNAs from Circm6A were included, Circm6A still achieve high F1 score (0.89) on detection of m^6^A-circRNAs (Fig. 2C and Additional file 1: Table S5).

For the strategy of RNA fragmentation before MeRIP, we also simulated three MeRIP-seq datasets with sequencing depth range from 60 to 80 million reads. This strategy is a frequently used approach in the m^6^A profiling studies. Compared to the strategy of RNA fragmentation after MeRIP, fragmentation of RNA before MeRIP will obtain higher resolution m^6^A sites. However, this strategy is not able to identify m^6^A-circRNAs with m^6^As distal to BSJ sites, since the sequenced fragments of these BSJ-distal m^6^A-circRNAs pulled down by m^6^A antibody do not contain BSJ site, which is the critical signal for detecting m^6^A-circRNAs needed by both Circm6A and other circRNA detection tools (Additional file 1: Fig. S1A). By applying Circm6A and other known tools to our simulated MeRIP-seq dataset, we indeed found that Circm6A and other known tools performed poorly in identifying m^6^As distal to BSJ sites (called “BSJ-distal m^6^A-circRNAs”) (F1 score < 0.01). However, Circm6A had much better performance in detecting m^6^As near BSJ (called “BSJ-proximal m^6^A-circRNAs”) than other tools (Circm6A: F1 score > 0.9; other tools: F1 score 0.28 ~ 0.74) (Fig. 2D).

To evaluate the depth of sequencing required for the identification of m^6^A-circRNAs, we simulated the MeRIP-seq data with sequencing depth from 1 M to 100 M and applied Circm6A to these simulated MeRIP-seq datasets. We found at sequencing depth of 40–50 M, Circm6A achieved satisfactory performance in the identification of both circRNA and m^6^A-circRNA (Fig. 2E). In addition, the time consumption of Circm6A was low (Additional file 1: Fig. S1C), and the Memory usage of Circm6A was approximately 32 GB (which can be set freely) (Additional file 1: Fig. S1D). Circm6A was implemented in JAVA and user-friendly.

Taken together, these results showed that Circm6A could accurately detect both BSJ-proximal and BSJ-distal m^6^A-circRNAs from MeRIP-seq data using the strategy of fragmentation of RNA after MeRIP, while could only accurately detect BSJ-proximal m^6^A-circRNAs from MeRIP-seq data using the strategy of fragmentation of RNA before MeRIP.

### The identification, validation, and characterization of m^6^A-circRNAs in PDAC tissue samples

**Fig. 3:**
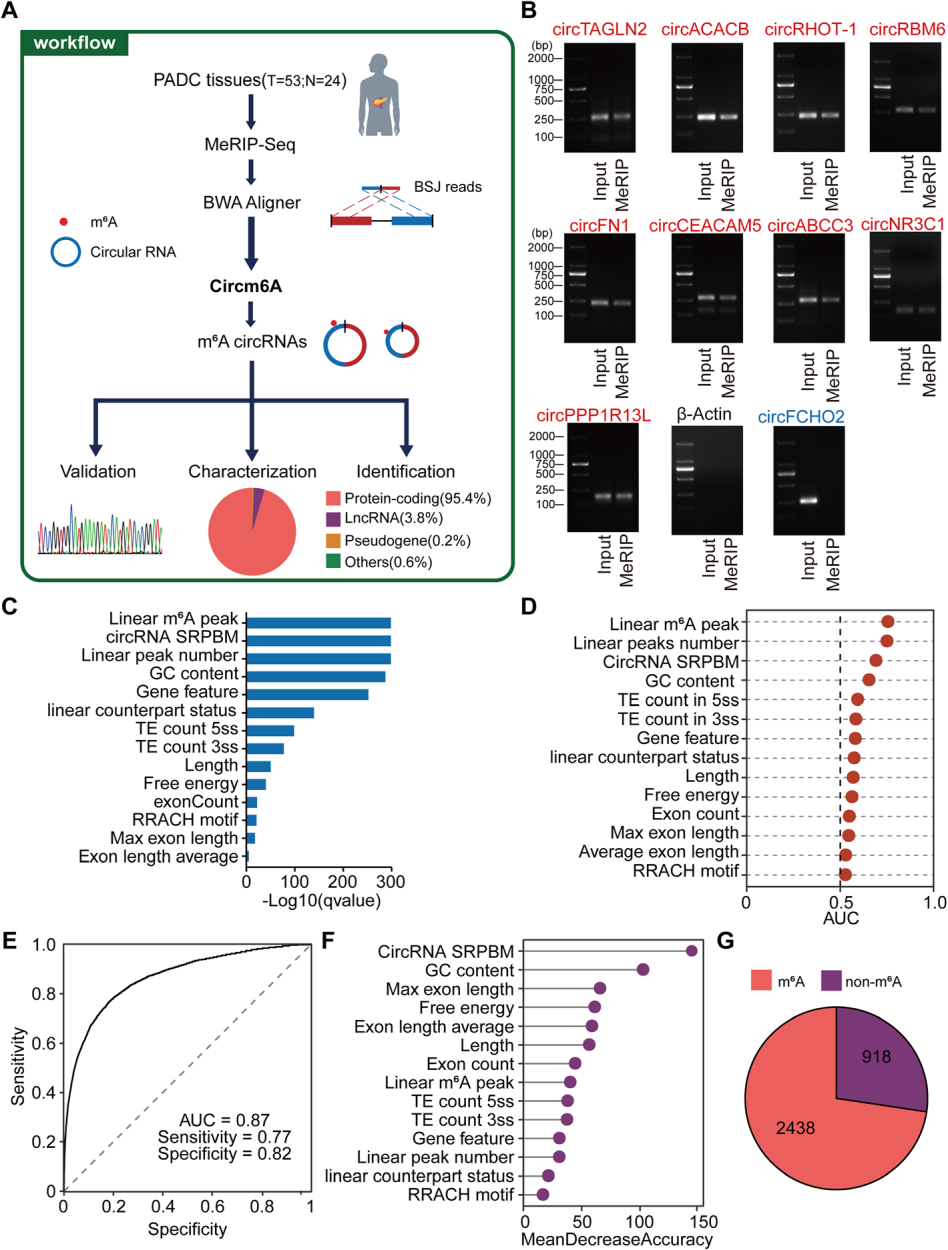
Identification and characterization of m^6^A-circRNAs in PDAC. **A** Workflow for the identification and characterization of m^6^A-circRNAs from MeRIP-seq of 77 tissue samples from 53 PDAC patients using Circm6A. **B** Validate the m^6^A modification of 9 m^6^A-circRNAs using MeRIP-qPCR after RNase R treatment. **C** Differential analysis results for 14 features for predicting m^6^A-circRNAs. Bars show FDR-corrected *P* values (*q* value). *P* values for quantitative features were calculated with the Wilcoxon rank sum test, and *P* values for qualitative variables were calculated with Pearson’s chi-squared test. **D** The power of prediction of m^6^A-circRNAs for the 14 features. **E** Receiver operating characteristic (ROC) curve for the random forest model in ten-fold cross-validation. **F** Mean decrease accuracy (MDA) for each feature in the random forest model. **G** The predicted result for the m^6^A status of “ambiguous circRNAs” by the random forest model. m^6^A, “ambiguous circRNAs” predicted as m^6^A-circRNAs; non-m^6^A, “ambiguous circRNAs” predicted as non-m^6^A circRNAs

We performed MeRIP-seq using fragmentation before MeRIP on the total RNAs from 77 samples from 58 PDAC patients, including 53 tumor and 24 normal tissue samples (Additional file 1: Table S1), and then applied Circm6A to these MeRIP-seq data (Fig. 3A). We detected a total of 38,164 circRNAs in all these samples (Additional file 2: Table S6), and among these circRNAs, 13,935 (36.5%), 12,634 (33.1%), and 5564 (14.6%) were recorded in the MiOncoCirc (PAAD cohort) [22], circBase [53], and CircRic [54] databases, respectively (Additional file 1: Fig. S2A). Moreover, the distribution of host gene features was similar to that of the circRNAs in the two public databases (Additional file 1: Fig. S2B, C), and the majority of circRNAs were flanked by the canonical splicing motif AG-GT, as were those from the two public databases (Additional file 1: Fig. S2D). We selected 9 circRNAs for further experimental validation, and 100% of circRNAs were validated by qRT-PCR in the PDAC samples (Additional file 1: Fig. S3A and Additional File 4). The back spliced exon-exon junctions of six circRNA candidates were also validated by Sanger sequencing (Additional file 1: Fig. S3B). These results suggested the reliability of Circm6A in the identification of circRNAs.

Among all these circRNAs identified from Circm6A in our PDAC MeRIP-seq data, Circm6A detected 6369 m^6^A-circRNAs. We selected 9 m^6^A-circRNAs and 1 non-m^6^A-circRNA for experimental validation using MeRIP-qPCR of the PDAC samples after RNase R treatment. All 9 m^6^A-circRNAs were found m^6^A-modified, and the non-m^6^A-circRNA did not harbor m^6^A modification (Fig. 3B and Additional File 4), further suggesting high accuracy of Circm6A. However, the 6369 m^6^A-cirRNAs detected from Circm6A were mainly BSJ-proximal m^6^A-circRNAs, due to the limitation of Circm6A in detecting m^6^A-circRNAs using the classic MeRIP-seq technology. During the library building of classic MeRIP-seq, the m^6^A modifications and their BSJ sites are likely to be separated during RNA fragmentation (Additional file 1: Fig. S1A), which will result in the miss of the BSJ-distal m^6^A-circRNAs. To retrieve those missed BSJ-distal m^6^A-circRNAs, we next built a machine learning model to predict them. “Potential BSJ-distal m^6^A-circRNA” are defined as non-m^6^A-circRNA whose genomic region exists a m^6^A modification in the region distal to its BSJ sites. Accordingly, we found 3356 “potential BSJ-distal m^6^A-circRNAs” that have linear m^6^A peaks located in their BSJ-distal region in our PDAC MeRIP-seq data. The machine learning model was then constructed using the well-defined m^6^A-circRNAs and non-m^6^A-circRNAs as the training set. We found that 14 features, such as exon count, exon length, circRNA level, circRNA length, m^6^A motif, and GC content, were significantly different between the m^6^A-circRNAs and non-m^6^A-circRNAs (Fig. 3C) and these 14 features showed ability of distinguishing m^6^A-circRNAs from non-m^6^A-circRNAs (Fig. 3D). Based on these 14 significant features, we constructed a model using random forest algorithm to predict the m^6^A modification of circRNA. Ten-fold cross-validation of the prediction model achieved a high AUC (0.87) (Fig. 3E). Among the 14 features, circRNA level and GC content were the two most important factors contributing to accuracy (Fig. 3F). We then applied our random forest model to predict the m^6^A modification of “potential BSJ-distal m^6^A-circRNAs”. Among 3356 “potential BSJ-distal m^6^A-circRNAs,” the majority (2438, 72.6%) were predicted as m^6^A-circRNAs (Fig. 3G). We considered the 2438 m^6^A-circRNAs predicted by random forest model as BSJ-distal m^6^A-circRNAs. We next integrated the 8807 BSJ-distal and BSJ-proximal m^6^A-circRNAs for the further analysis.

### Dysregulation of m^6^As in circRNAs and their functional significance in PDAC

**Fig. 4:**
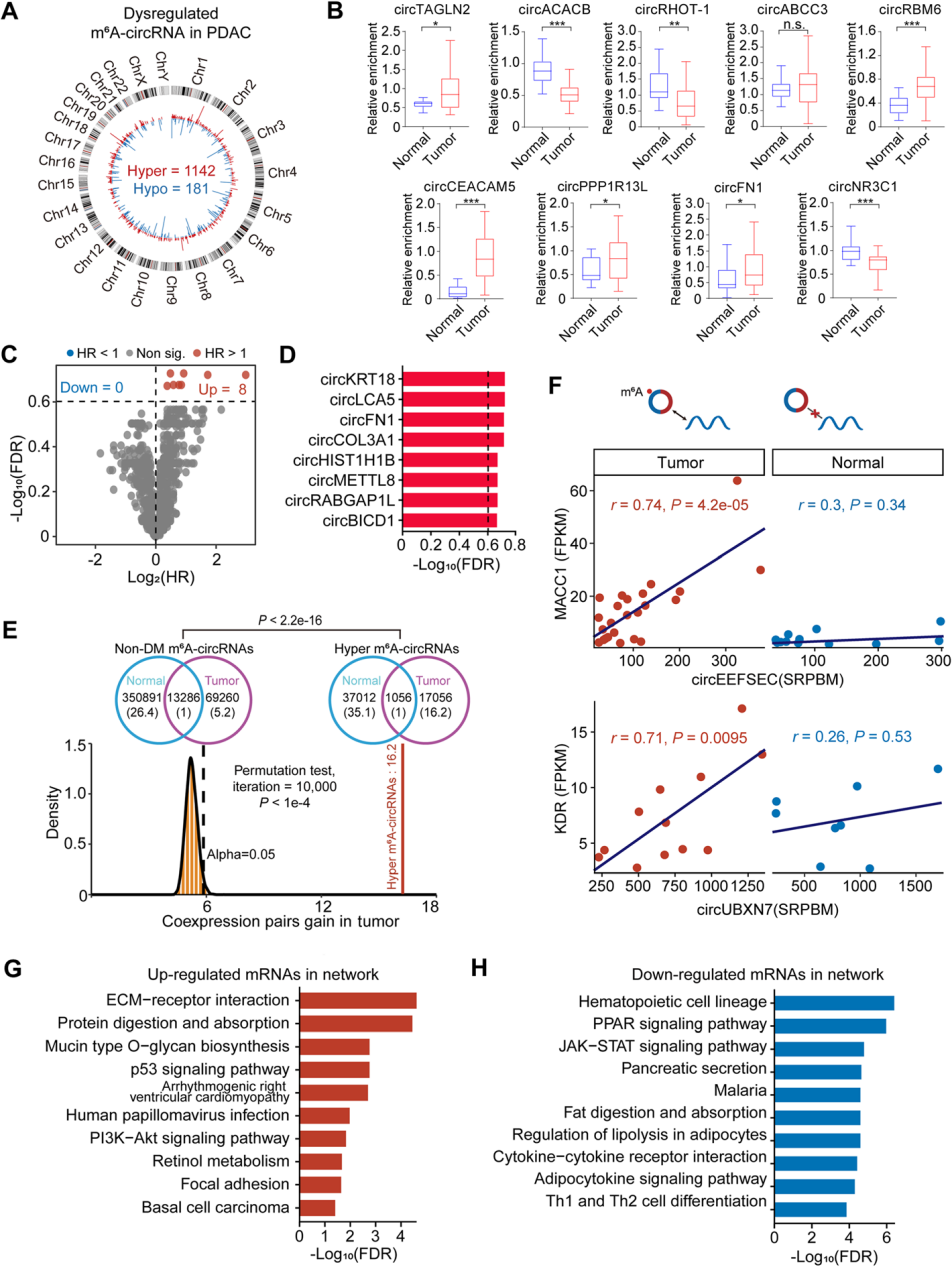
Differential m^6^A methylation of m^6^A-circRNAs between PDAC tumor and normal tissues**. A** The circos plot shows the genome-wide hypermethylated (red bar) and hypomethylated (blue bar) circRNAs in PDAC tumor tissues. The height of the bar represents the frequency of m^6^A modification in all samples. **B** Validation of differentially methylated m^6^A-circRNAs using MeRIP-qPCR. *, *P* < 0.05; **, *P* < 0.01; ***, *P* < 0.001; n.s. P > 0.05 of Student’s t test. **C, D** The correlation between PFS and m^6^A level of differentially methylated m^6^A-circRNAs in PDAC. Volcano plot depicting log_2_ hazard ratios (HRs) and − log_10_ (FDR) values of differentially methylated m^6^A-circRNAs in the Cox proportional hazards model of PFS. The horizontal dashed line in **C** and vertical dashed line in **D** correspond to “FDR = 0.25”. All 8 differentially methylated m^6^A-circRNAs significantly associated with PFS are showed in **D**. **E** The number of coexpressed pairs for hypermethylated m^6^A-circRNAs and non-differentially methylated m^6^A-circRNAs (non-DM m^6^A-circRNAs) in PDAC tumor and normal tissue samples, respectively (upper panel). The number in parentheses is normalized to the number of coexpressed pairs shared between the tumor and normal samples. The permutation test shows significant contribution of hypermethylated m^6^A-circRNAs to the gain of coexpression network in tumor tissue samples compared with randomly selected non-DM m^6^A-circRNAs (lower panel). Vertical red lines indicate the gain fold of hypermethylated m^6^A-circRNAs, the histogram indicates the fold gain of randomly selected non-DM m^6^A-circRNAs. **F** Scatter plots showing the coexpression of circEEFSEC-MACC1 pair (upper panel) and circUBXN7-KDR pair (lower panel) in PDAC tumor and normal tissue samples, respectively. *r* represents Pearson correlation coefficients; *P* represents *P* value. **G, H** Top enriched KEGG pathways for upregulated mRNAs (red, **G**) and downregulated mRNAs (blue, **H**) in the coexpression network that hypermethylated m^6^A-circRNAs were involved in

We found that among the 8807 m^6^A-circRNAs, 1142 were hypermethylated while only 181 m^6^A-circRNAs were hypomethylated in tumor tissues compared with normal tissues (Fig. 4A and Additional file 2: Table S7). Eight out of 9 selected hyper/hypomethylated m^6^A-circRNAs could be validated by MeRIP qRT-PCR, further suggesting the reliability of the results (Fig. 4B). We found some hyper/hypomethylated m^6^A-circRNAs have clinical significance. After multiple hypothesis correction, 8 dysregulated m^6^A-circRNAs showed a negative correlation (FDR ≤ 0.25) with progression-free survival (PFS) (Fig. 4C, D) and 22 dysregulated m^6^A-circRNAs showed a negative correlation (FDR ≤ 0.25) with overall survival (OS) (Additional file 1: Fig. S4A, B).

It has been reported that circRNAs could regulate mRNAs by competing RNA-binding proteins or acting as scaffolds for RNA-binding proteins [55, 56]. We therefore suspected that m^6^A-circRNAs will regulate the expression of mRNAs by recruiting the m^6^A writers/readers/erasers through their m^6^A sites. To explore this, we firstly constructed a circRNA/mRNA coexpression network (detail in “Methods” section) by using all circRNAs and differentially expressed mRNAs between PDAC tumor and normal tissues (1206 upregulated and 1117 downregulated mRNAs were detected in PDAC tumor tissues, as shown in Additional file 1, Fig. S4C). Intriguingly, m^6^A-circRNA/mRNA pairs showed a significantly higher correlation than non-m^6^A-circRNA/mRNA pairs after eliminating the interference of microRNA (Wilcoxon rank sum test, *P* = 3.14e−13), suggesting that m^6^A can affect the coexpression between the circRNAs and the mRNAs in PDAC. Moreover, hypermethylated m^6^A-circRNAs were widely associated with a gain of coexpression network in PDAC tumor tissues, while nondifferentially methylated m^6^A-circRNAs were not (16.2- versus 5.2-fold gain, Pearson’s chi-squared test *P* < 2.2e−16, permutation test *P* < 1e−4) (Fig. 4E). In total, 671 hypermethylated m^6^A-circRNAs resulted in a gain of mRNA-circRNA coexpression in PDAC tumor tissues (Additional file 3: Table S8). For example, the hypermethylated m^6^A in circ*EEFSEC* and circ*UBXN7* resulted in the gain of coexpression of circ*EEFSEC-MACC1* (*r* = 0.74, *P* = 4.2e−5) and circ*UBXN7*-*KDR* (*r* = 0.71, *P* = 0.0095) in tumor samples and while there was no correlation between circ*EEFSEC* and *MACC1* (*r* = 0.3, *P* = 0.34) and between circ*UBXN7* and *KDR* (*r* = 0.25, *P* = 0.53) in normal samples. Moreover, the gains of coexpression in PDAC tumor were also observed in circ*ITGB6-MECOM* and circ*PTK2-ETV1* (Fig. 4F and Additional file 1: Fig. S4D). The 1029 upregulated mRNAs that were involved in the aberrantly methylated m^6^A-circRNA/mRNA coexpression network were mainly enriched in cancer-related pathways, such as the ECM-receptor interaction, mucin type O-glycan biosynthesis, and p53 signaling pathway, while the 925 downregulated mRNAs that were involved in this network were mainly enriched in pathways related to PPAR signaling pathway and Th1 and Th2 cell differentiation (Fig. 4G, H). These results suggested that the dysregulation of m^6^As in circRNAs might have critical functional significance in PDAC.

We speculated that m^6^A-dependent crosstalk between circRNAs and mRNAs in the coexpression network was probably mediated by m^6^A readers, as m^6^A functions through recruiting readers. Indeed, we found that all reported m^6^A readers significantly preferred to bind the comethylated pairs (both the circRNA and mRNA were m^6^A methylated in the coexpression network) (Additional file 1: Fig. S4E), and YTHDF1 and YTHDF2 were correlated with a significant proportion of differentially expressed mRNAs involved in the coexpression network (Additional file 1: Fig. S4F), indicating that these two “readers” may play a role in m^6^A-dependent crosstalk.

### m^6^A modification is associated with the circularization of circRNAs

**Fig. 5:**
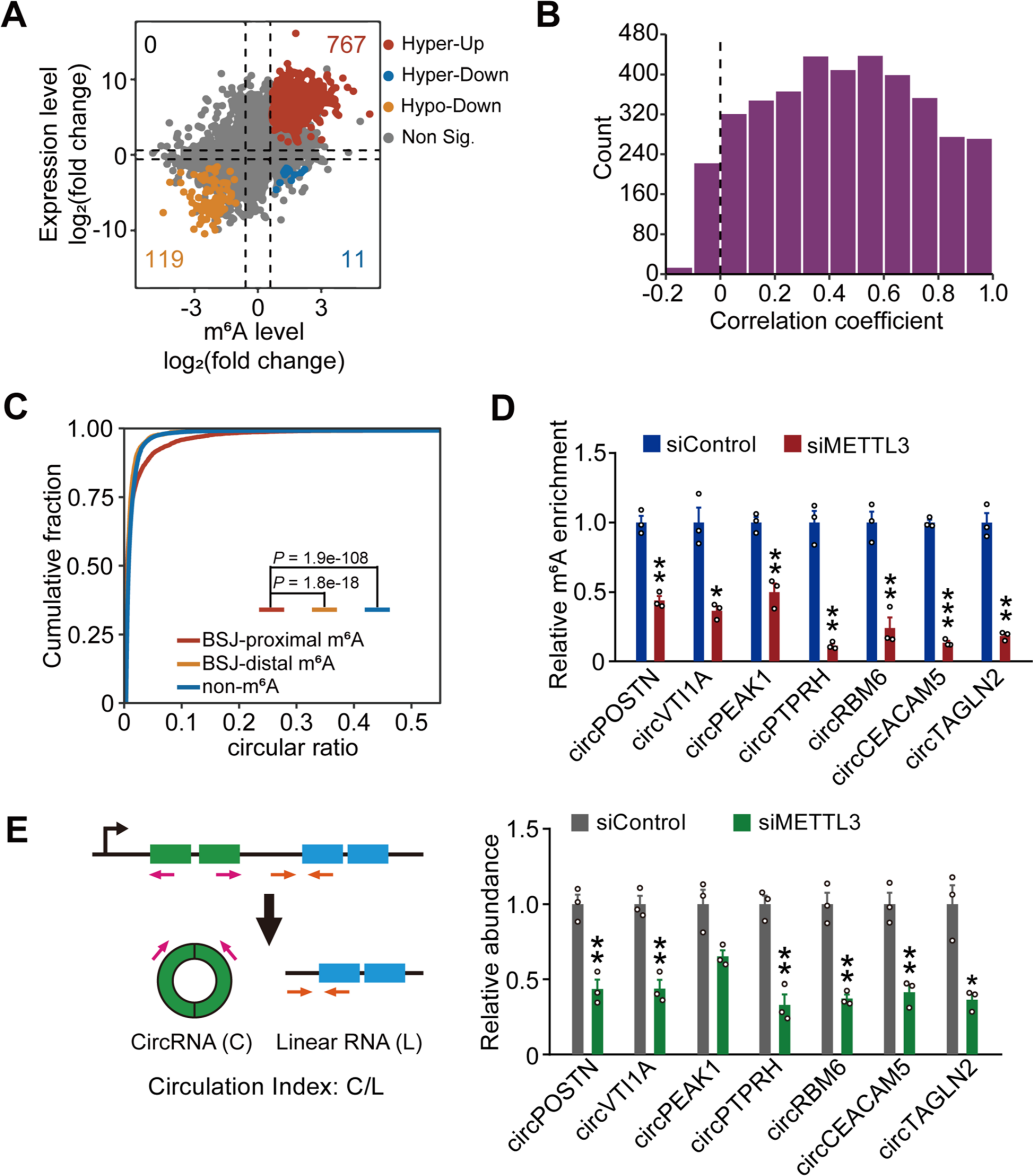
m^6^A modification is associated with the circularization of circRNAs. **A** Distribution of m^6^A-circRNAs with a significant change in both the m^6^A level and circRNA expression level. **B** The distribution of correlation coefficients (Pearson correlation coefficient) between the m^6^A level and circRNA expression level. **C** Cumulative fraction of the circular ratio for m^6^A-circRNAs and non-m^6^A-circRNAs, respectively. The *P* value was calculated with the Wilcoxon rank sum test. **D** MeRIP-qPCR analysis of m^6^A levels of 7 indicated circRNAs in PDAC cell line with METTL3 knockdown and control. **E** Circulation index of 7 indicated circRNAs in PDAC cell line with METTL3 knockdown and control (bottom panel). Circulation index represent the relative abundance of each circRNA to its pre-mRNA. The abundance of circRNA and its pre-mRNA were determined using qRT-PCR. Top panel showed the scheme of primer design for circRNA and linear RNA to calculate circularization index. “C” and “L” primer sets are used to quantify circRNAs and linear RNAs. Data in **D** and **E** were means ± S.E.M. (*n* = 3). *, *P* < 0.05; **, *P* < 0.01; ***, *P* < 0.001 of Student’s *t* test

We next examined whether there is any effect of m^6^A modification on circRNA abundance. Interestingly, we found 85.5% (767/897) of circRNAs show the same direction of change between tumor and normal tissue samples at m^6^A and circRNA expression level, meaning hypermethylated m^6^A-circRNAs tended to upregulate circRNA expression in PDAC (hypermethylated/upregulated circRNAs, 767), and hypomethylated m^6^A-circRNAs tended to downregulate circRNA expression (hypomethylated/downregulated, 119) (Fig. 5A). We experimentally validated the differential expression of 9 selected candidates (Additional file 1: Fig. S5A). Further integrated analysis revealed positive correlations between the m^6^A level and the circRNA level (Fig. 5B). As previously reported, m^6^A can regulate the stability of transcripts by recruiting “readers,” such as YTHDF and IGF2BP proteins [3, 57, 58]. We first examined the correlation between the expression level of m^6^A readers and circRNAs. However, we found that there were few hypermethylated-upregulated circRNAs significantly correlated with these reported “readers,” indicating that m^6^A modification in circRNAs might not enhance expression level of circRNAs via recruiting “readers” in pancreatic cancer (Additional file 1: Fig. S5B). Since the circRNA level may also be regulated by circularization, we further investigated the relationship between m^6^As and the circular ratio of circRNAs. Interestingly, we found that BSJ-proximal m^6^A-circRNAs displayed a significantly higher circular ratio than BSJ-distal m^6^A-circRNAs and non-m^6^A-circRNAs (Fig. 5C), suggesting m^6^As locating near BSJ sites play critical role in the formation of circRNAs. The circularization process is regulated by multiple factors [6], such as ADAR (double-stranded RNA-specific adenosine deaminase) [59]. Closer inspection revealed that flanking regions of m^6^A peak centers were less occupied by ADAR1 compared to background (Additional file 1: Fig. S5C), further suggesting that m^6^As are related to the formation of circRNAs. To further validate our result, 4sU pulse labeling and nascent RNA collection were then performed after METTL3 knockdown (Additional file 1: Fig. S5D) to measure circularization index of circRNAs (CI, the relative abundance of circRNA [C] versus spliced linear mRNA [L]) of circRNA-generating genes. In agreement with the sequencing results, the decreased m^6^A level of examined circRNAs significantly reduced their circularization efficiency (Fig. 5D, E). These observations suggested that m^6^A modifications contribute to the biogenesis of circRNAs.

### Hyper-m^6^A-circRNAs possess potential for translation

**Fig. 6:**
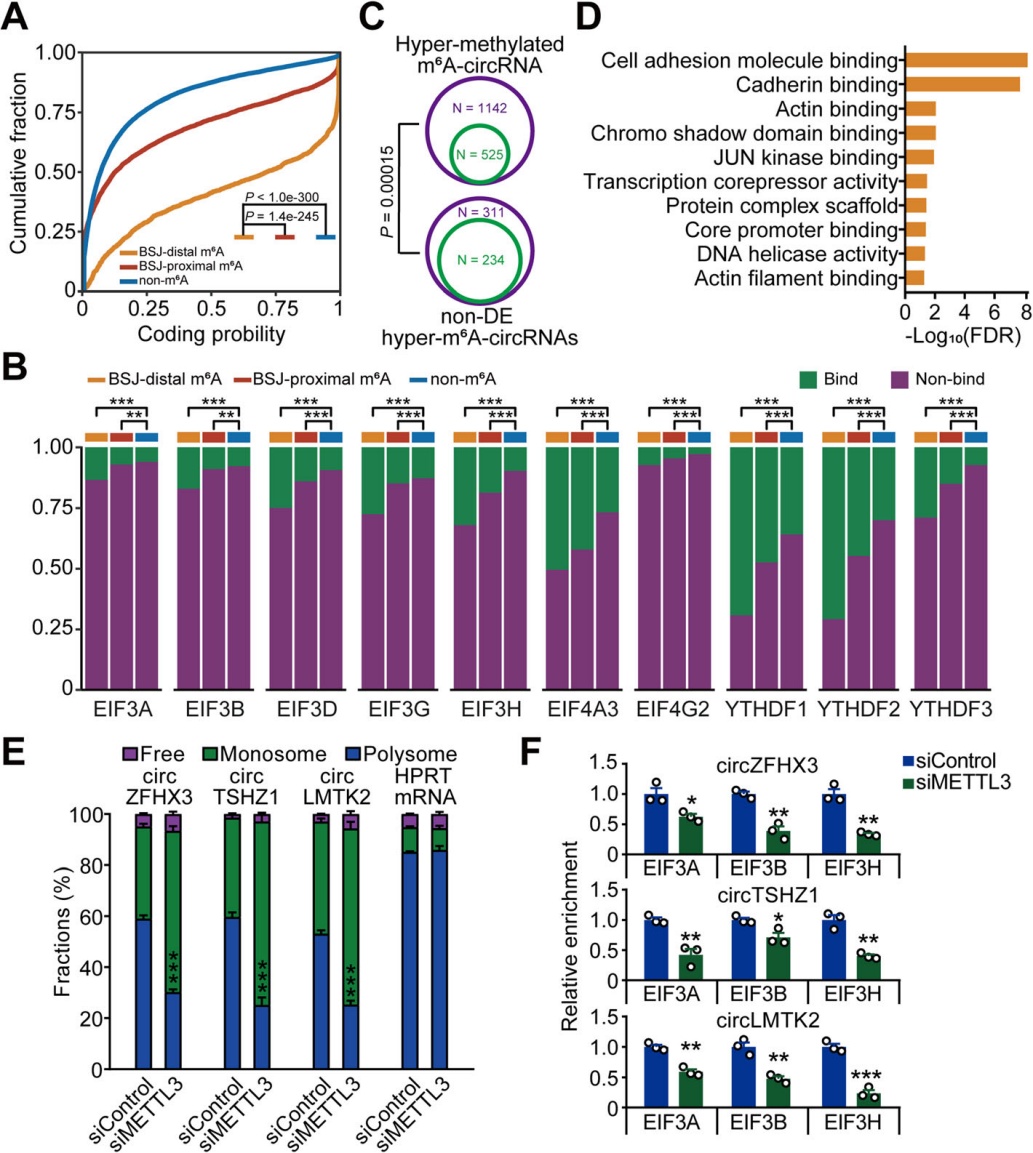
The hypermethylated m6A-circRNAs possess potential for translation. A The cumulative fraction of coding probability scores predicted by CPAT for BSJ-distal m6A-circRNAs, BSJ-near m6A-circRNAs and non-m6A-circRNAs. The P value was calculated with the Wilcoxon rank sum test. B Significant higher ratio of binding sites of EIFs and YTHDFs in BSJ-distal m6A-circRNAs than BSJ-near m6A-circRNAs and non-m6A-circRNAs. For each clustered bar, from left to right were binding sites ratios of BSJ-distal m6A-circRNAs, BSJ-near m6A-circRNAs, and non-m6A-circRNAs. P values were calculated by chi-squared test; *, P < 0.05; **, P < 0.01; ***, P < 0.001. C Venn diagram showing the number of m6A-circRNAs with coding potential (circRNAs with ORFs and EIFs binding sites) for non-DE hypermethylated-m6A-circRNA and all hypermethylated-m6A-circRNA. The outer layer indicated total number of corresponding m6A-circRNAs; the inner layer indicated the number of corresponding m6A-circRNAs with coding potential. Fisher’s exact test was used to derive the P value. D GO molecular function (MF) terms enrichment analysis for host genes of hypermethylated m6A-circRNAs with coding potential. E Relative fractions of unbound (free) RNAs, monosome- and polysome-bound RNAs for circZFHX3, circTSHZ1, and circLMTK2 in PDAC cell lines with METTL3 siRNA and control. The HPRT mRNA was used as control. F RIP-qPCR analysis showed EIF3A-, EIF3B-, and EIF3H-bound RNA abundance for circZFHX3, circTSHZ1, and circLMTK2 in PDAC cells with or without METTL3 knockdown. Data in E and F were means ± S.E.M. (n = 3). *, P < 0.05; **, P < 0.01; ***, and P < 0.001 of Student’s t test comparing with each control

The m^6^A modification is reported to regulate the translation of circRNAs [13]. We next dedicated to explore the relationship between m^6^A modification and circRNA translation in the identified m^6^A-circRNAs in PDAC samples. To this end, we firstly predicted coding potential using Coding potential assessing tool (CPAT) [60] and found that both BSJ-distal and BSJ-proximal m^6^A-circRNAs displayed significantly higher coding probability scores than non-m^6^A-circRNAs (*P* < 2.2e−16; Wilcox rank sum test). Interestingly, BSJ-distal m^6^A-circRNAs had significant higher coding probability scores than BSJ-proximal m^6^A-circRNAs (Fig. 6A). Moreover, we found BSJ-distal m^6^A-circRNAs had longer open reading frames (ORFs) compared with BSJ-proximal m^6^A-circRNAs and non-m^6^A-circRNAs (Additional file 1: Fig. S6A). To further investigate the effects of m^6^A on the translation of circRNAs, we systematically analyzed the binding sites within circRNAs for translation-related m^6^A “readers” (such as YTHDF1, YTHDF3) and eukaryotic initiation factors (EIFs) that were previously reported to promote translation [13, 61]. We found that the ratio of binding sites for EIFs (EIF3A, EIF3B, EIF3D, EIF3G, EIF3H, EIF4A3, EIF4G2) and YTHDF1-3 were more enriched in BSJ-distal m^6^A-circRNAs than that in BSJ-proximal m^6^A-circRNAs and non-m^6^A-circRNAs (Fig. 6B), which was further confirmed by permutation tests (Additional file 1: Fig. S6B). These results suggested that m^6^A-circRNAs are more likely to have translational capacity than non-m^6^A-circRNAs, and among these m^6^A-circRNAs, BSJ-distal m^6^A-circRNAs showed stronger translational capacity than BSJ-proximal m^6^A-circRNAs.

Interestingly, we found that the percentage of circRNAs harboring binding sites for EIFs (EIF3A, EIF3B, EIF3D, EIF3G, EIF3H, EIF4A3, EIF4G2) in nondifferentially expressed (non-DE) hyper-m^6^A-circRNAs was significantly higher compared to all hyper-m^6^A-circRNAs (Fisher’s exact test, *P* = 0.00015) (Fig. 6C), indicating that m^6^A modifications of some hyper-m^6^A-circRNAs might not be functional through affecting circRNA formation, but through affecting translation. To elucidate the biological modules those hyper-m^6^A-circRNAs potentially affecting translation (hyper-m^6^A-circRNAs with ORFs and EIFs/YTHDFs binding), we carried out GO enrichment analysis on their host gene. We found that hyper-m^6^A-circRNAs enrich in GO molecular function (MF) terms on transcription process such as core promoter binding and transcription core repressor activity (Fig. 6D). We selected several circRNAs involved in these GO term for further experiment validation. After knockdown of METTL3, the m^6^A level and translation activity of circZFHX, circTSHZ1 and circLMTK2 were significantly reduced (Fig. 6E and Additional file 1: Fig. S6C, D). We further observed that knockdown of METTL3 attenuated the binding of EIFs (EIF3A, EIF3B and EIF3H) to these hyper-m^6^A-circRNAs (Fig. 6F). These findings indicated that the hypermethylation of some m^6^A-circRNAs might cause the dysregulation of circRNA translation.

## Discussion

In recent years, both circRNAs and m^6^A modifications have attracted increasing attention in cancer studies due to their important roles in cancer progression in various types of cancer [9, 11, 22, 62–64]. However, few studies have brought together the two rapidly expanding fields in cancer studies. In particular, transcriptome-wide mapping of m^6^A modifications in circRNAs in cancer patients is still lacking. In this study, we developed a de novo algorithm for the transcriptome-wide mapping of m^6^A modifications in circRNAs from m^6^A-seq data and applied this algorithm to the m^6^A-seq data from a large sample of individuals with PDAC with the goal of exploring the function and regulation of m^6^A-circRNA in cancer.

Our de novo algorithm has several advantages compared to the existing methods for transcriptome-wide mapping of m^6^A modifications in circRNAs [12, 13]. Zhou et al. considered that all the circRNAs detected from m^6^A IP samples have potential m^6^A modifications [12]. However, antibodies recognizing m^6^A will also capture RNAs without m^6^A modifications in a nonspecific manner [65, 66]; therefore, the detection of circRNA signals only in IP samples without considering the background noise as presented in INPUT samples will probably introduce false positives of m^6^A-circRNAs. Yang et al. employed m^6^A IP for RNA samples treated with exoribonuclease RNase R (circRNA-m^6^A-seq) to specifically identify m^6^A modifications in circRNAs [13]. Similar to the methods of Zhou et al., circRNA-m^6^A-seq also does not consider the nonspecific precipitation of RNAs by antibodies targeting m^6^A. Moreover, circRNA-m^6^A-seq does not detect the linear transcripts and their m^6^A modification; thus, crosstalk between circRNAs and mRNAs cannot be well explored. To address these issues, we developed a de novo algorithm called “Circm6A” to detect m^6^A modification in circular RNAs and linear transcripts from m^6^A-seq data. We performed Fisher’s exact test on the circRNA signal difference between IP and INPUT samples to determine the probability of observing a circRNA signal in IP samples caused by nonspecific capture.

However, Circm6A still has limitations when being applied to the MeRIP-seq data that was generated using the strategy of fragmentation before m^6^A-IP, which is the more frequently used library construction strategy since it could detect higher resolution m^6^A sites compared to the strategy of fragmentation after m^6^A-IP. For the strategy of fragmentation before m^6^A-IP, if the m^6^As are located in the circRNAs but distal to BSJs, the circRNA fragments pulled down by m^6^A-IP will not have BSJ signal thus the m^6^A enrichment signal will not be connected with the circRNAs. Therefore, the m6A-circRNAs with m6As distal to BSJs will not be detected by Circm6As and other circRNA detection tools. The analysis on our simulated data validated this assumption. To address this limitation, we developed a random forest model for the prediction of m^6^A modification status from the MeRIP-seq data. The random forest model will complement the Circm6A results.

We described a comprehensive landscape of m^6^A modification in circRNA in PDAC by using Circm6A. The m^6^A modifications in many circRNAs were dysregulated in PDAC tumor tissues compared to adjacent normal tissues, suggesting their functional roles in cancer progression. The identification of dysregulated m^6^A-circRNAs expanded our understanding of the biological role of m^6^A modification in cancer. Previous studies have reported that circRNAs function by regulating the metabolism of linear mRNA [8, 21, 67, 68]. We therefore explored the function of circRNAs and their m^6^A modifications in a circRNA-mRNA coexpression network. Intriguingly, we found that m^6^A modifications had a positive effect on circRNA-mRNA coexpression, and hypermethylated circRNAs significantly amplified the circRNA-mRNA coexpression network in PDAC tumor tissues compared to normal tissues. CircRNAs are intensively reported to function as miRNA sponges to regulate linear mRNA expression [8, 69, 70]. A few studies have reported that circRNAs could also modulate linear mRNAs by competing with RNA-binding proteins or acting as scaffolds for RNA-binding proteins [55, 56]. We propose several potential mechanism models for m^6^A-dependent crosstalk. One possible explanation is that m^6^A-circRNAs perhaps serve as sponges to sequester m^6^A readers from linear mRNAs with m^6^As, thereby reducing the effect of m^6^A readers on linear mRNAs. The other possible mechanism might be that m^6^A-circRNAs might function as scaffolds that promote the binding of readers to linear mRNAs. Indeed, Chen et al. have reported that circNSUN2, an m^6^A-circRNA, can enhance the stability of *HMGA2* mRNA by forming a circNSUN2/IGF2BP2/HMGA2 RNA-protein ternary complex [14].

m^6^A modifications have critical roles in many aspects of RNA regulation, including RNA stability, splicing, and translation [2, 3, 71–74]. In this study, we found that the hypermethylation of circRNAs tended to elevate circRNA levels in PDAC tumor tissues compared to normal tissues, and m^6^A levels were significantly positively correlated with circRNA abundance, whereas we observed that few circRNA were significantly coexpressed with well-known m^6^A readers that regulates RNA stability, suggesting that m^6^As might not regulate expression of circRNAs by recruiting “reader” to stabilize circRNAs. Apart from stability, the biogenesis of circRNA is another factor influencing circRNA levels. Interestingly, we found that m^6^A-circRNAs have a higher circular ratio than non-m^6^A-circRNAs, suggesting that m^6^As might play a potential role in the biogenesis of circRNAs. ADAR1, an RNA-editing enzyme, has been reported to suppress the biogenesis of circRNAs because A-to-I editing can diminish RNA pairing and thus prevent back-splicing for circRNA biogenesis [75]. Xiang et al. reported that ADAR1 is unfavorably associated with m^6^A-modified transcripts for further A-to-I editing and that m^6^A modification suppresses A-to-I editing on the same transcripts [76]. Consistently, we observed that the flanking region of m^6^A-circRNAs has fewer binding sites for ADAR1 than that of non-m^6^A-circRNAs, indicating a potential role of m^6^A in the regulation of circRNA biogenesis in which m^6^A modification suppresses A-to-I editing of pre-mRNAs and hence results in recovery of RNA pairing, eventually promoting the biogenesis of circRNAs; however, this mechanism requires further investigation. Previous researches have reported that some circRNAs have translational abilities [77–81]. Moreover, m^6^A is reported to promote the translation efficiency of circRNAs [13].

## Conclusions

In conclusion, we firstly developed a computational tool, Circm6A, to detect m^6^A modification in circRNAs from m^6^A-seq data specially, which would facilitate following researches on m^6^A-circRNAs. With application of Circm6A to our PDAC m^6^A-seq data, we showed the genomic landscape of m^6^A-circRNAs in PDAC patients for the first time. Further analysis showed that m^6^A-circRNAs tended to be hypermethylated in PDAC tumor tissues compared to adjacent normal tissues. Surprisingly, we found that hypermethylated m^6^As-circRNAs were related with the expansion of circRNA-mRNA coexpression network involved in many cancer-related pathways. Further, our bioinformatic analysis and experiment results also indicated that m^6^A modifications in circRNAs might promote the circularization and translation of circRNAs. These comprehensive findings shed new light on the regulatory perspective of m^6^A modification in circRNAs and the function importance of m^6^A-circRNAs in PDAC.

## Supplementary Information

**Additional file 1: Fig. S1 to Fig. S6 and Table S1 to Table S5.** Supplementary figures and Tables.

**Additional file 2: Table S6 and Table S7.** Supplementary Tables.

**Additional file 3: Table S8.** The gained coexpressed circRNA-mRNA pairs of hyper m^6^A-circRNAs in PDAC cancer tissues.

**Additional file 4.** uncropped blot for Fig. S3A and Fig. 3B.

## Data Availability

The bioinformatics tools Circm6A (https://github.com/canceromics/circm6a [82]) and MeRIP-simulator at (https://github.com/canceromics/MeRIP-simulator [83]) are both available for free online. Data analysis scripts in the study were deposited in github (https://github.com/finallyisnoone/Circm6A.git [84]). The raw sequencing data of this study have been deposited in the Genome Sequence Archive of Beijing Institute of Genomics, Chinese Academy of Sciences (https://ngdc.cncb.ac.cn), with accession number HRA000102 (https://ngdc.cncb.ac.cn/gsa-human/browse/HRA000102).

## Abbreviations

m^6^A: *N*^*6*^-methyladenosine
circRNA: Circular circRNA
BSJ: Back-splicing junction
PDAC: Pancreatic ductal adenocarcinoma
IP: Immunoprecipitation
MeRIP-seq: m^6^A-specific methylated RNA immunoprecipitation followed by high-throughput sequencing
m^6^A-circRNA: Circular RNA with m^6^A modification
non-m^6^A-circRNA: Circular RNA with m^6^A modification
qRT-PCR: Quantitative real-time PCR
PFS: Progression-free survival
OS: Overall survival
ORF: Open reading frames
F1 score: The harmonic mean of the precision and recall
FDR: False discovery rate
AUC: Area under the curve
GO: Gene ontology
