# Supplementary material for: Genome-wide identification and characterization of circular RNA m6A modification in pancreatic cancer
Source: Genome Med. 2021 Nov 19;13:183. doi: 10.1186/s13073-021-01002-w (PMC8605608; doi:10.1186/s13073-021-01002-w)
Supplement: Supplementary file 1 — Additional file 1: Fig. S1 to Fig. S6 and Table S1 to Table S5. Supplementary figures and Tables. [file 13073_2021_1002_MOESM1_ESM.pdf]

Additional file 1:

Supplementary Materials for

Genome-wide identification and characterization of circular RNA m^6A modification in pancreatic cancer

Ying Ye[1,†], Weiyi Feng[1,†], Jialiang Zhang[1,†], Kaiyu Zhu[1], Xudong Huang[1], Ling Pan[1], Jiachun Su[1], Yanfen Zheng[1], Rui Li[1], Shuang Deng[1], Ruihong Bai[1], Lisha Zhuang[1], Lusheng Wei[2], Junge Deng[1], Mei Li[3], Rufu Chen[4], Dongxin Lin[1,5,6,*], Zhixiang Zuo[1,*], Jian Zheng[1,6,*]

[†]These authors contributed equally to this work.

*Correspondence should be addressed to Prof. Jian Zheng (zhengjian@sysucc.org.cn), Prof. Zhixiang Zuo (zuozhx@sysucc.org.cn) or Prof. Dongxin Lin (lindx@sysucc.org.cn) at Sun Yat-sen University Cancer Center, Guangzhou, China

This PDF file includes:

Fig. S1 to Fig. S6

Table S1 to Table S5

Ye et al. Fig. S1

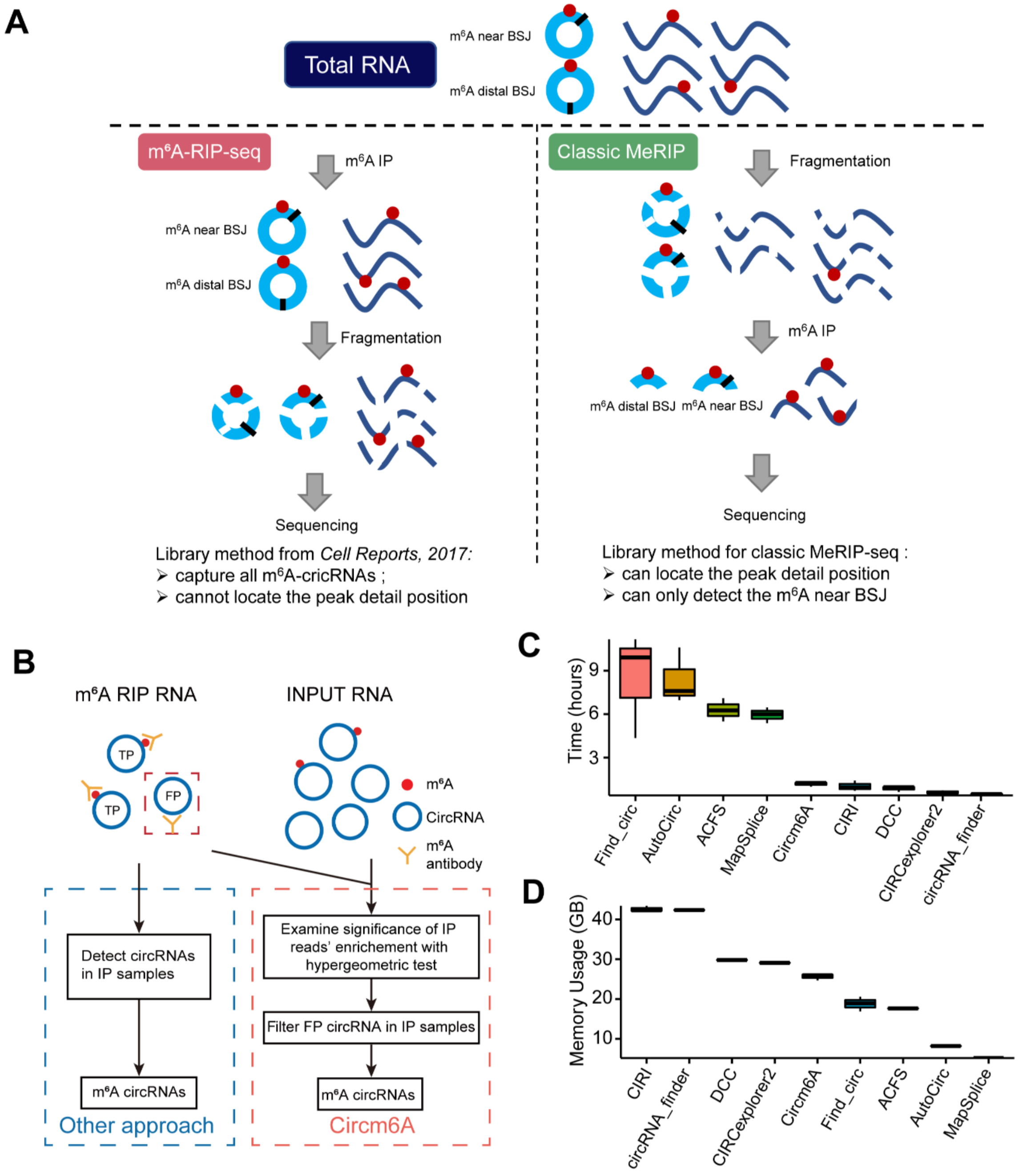

Fig. S1. Additional figures related to Fig. 2. (**A**) Two MeRIP-seq library construction strategies. Left panel shows strategy of fragmentation of RNA after m^6A antibody IP (MeRIP). Right panel shows strategy of fragmentation of RNA before MeRIP. (**B**) Left panel shows the m^6A-circRNA identification strategy that directly detected circRNAs in the m^6A IP sample and defined them as m^6A-circRNAs. Right panel shows the strategy used in Circm6A. TP, true positive; FP, false positive. (**C**) The Time consumption of Circm6A and other nine tools in predicting m^6A-circRNAs with simulated MeRIP-seq datasets (N = 3). (**D**) Memory usage of Circm6A and nine other tools.

Ye et al. Fig. S2

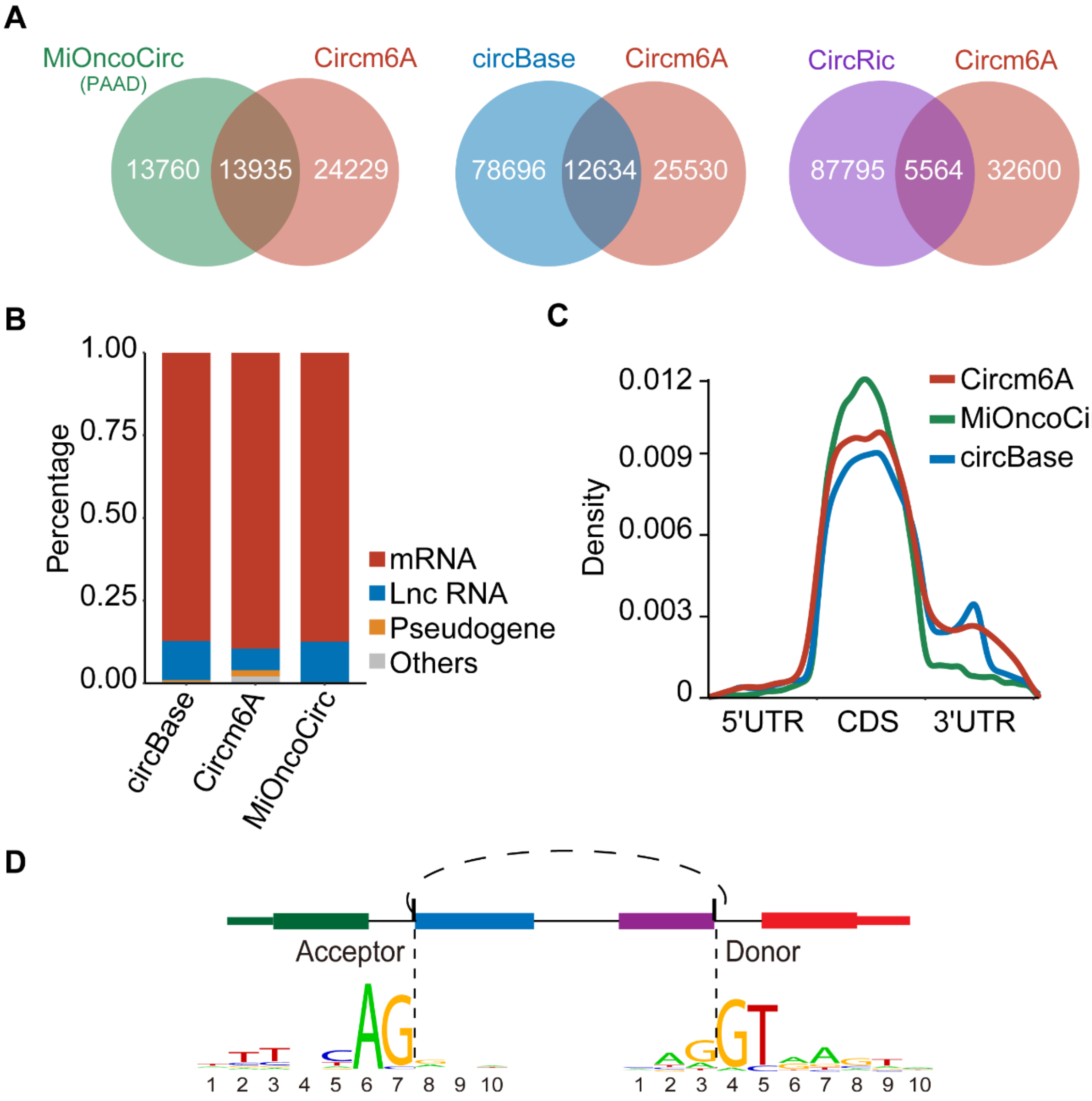

MiOncoCirc	AG	Not AG
GT	0.99	0.007
Not GT	0.004	0.002

Circm6A	AG	Not AG
GT	0.92	0.01
Not GT	0.03	0.04

CircBase	AG	Not AG
GT	0.78	0.05
Not GT	0.14	0.03

Fig. S2. Characteristics of circRNAs in PDAC. (**A**) Overlap of Circm6A-identified circRNAs with those circRNAs identified by the MiOncoCirc (from pancreatic cancer tissues), circBase databases and CircRic. (**B-C**) Distribution of host genes and the metagene plot for circRNAs in PDAC and the two known databases. (**D**) Upper panel: circular RNA was predominantly flanked by a canonical splicing motif, AG-GT (99.2%). Lower panel: statistics table for splicing motifs of circRNAs in PDAC and the two known databases.

Ye et al. Fig. S3

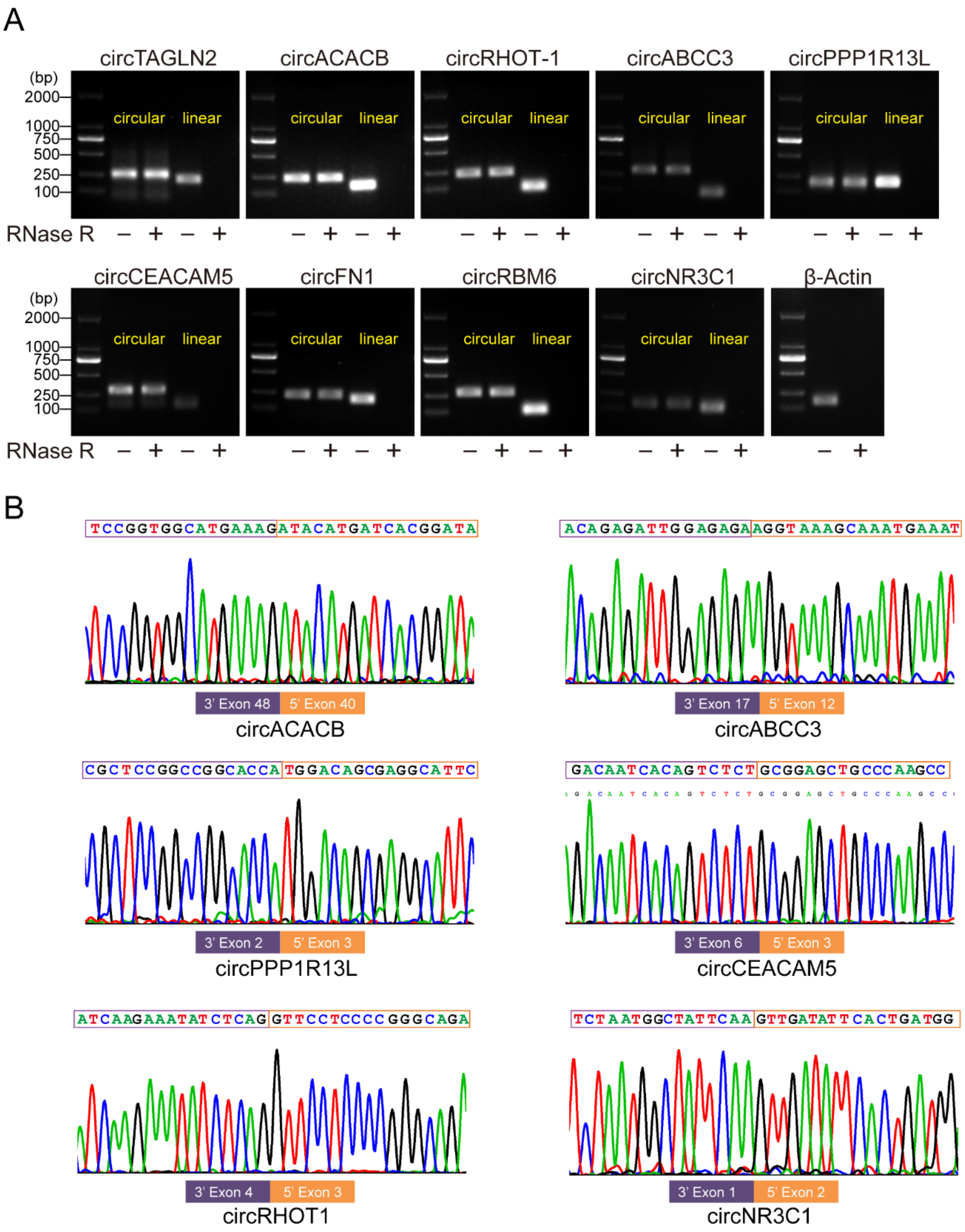

Fig. S3. Validation of circRNAs identified by Circm6A in PDAC. (**A**) Validation of 9 candidate circRNAs with qRT-PCR in PDAC tissue samples after RNase R treatment. (**B**) Sanger sequencing of the PCR product by divergent primers targeting the junction sites of the selected circRNAs to confirm back-splice sequences. The junction between the purple region and the orange region indicates the "head-to-tail" splicing site.

Ye et al. Fig. S4

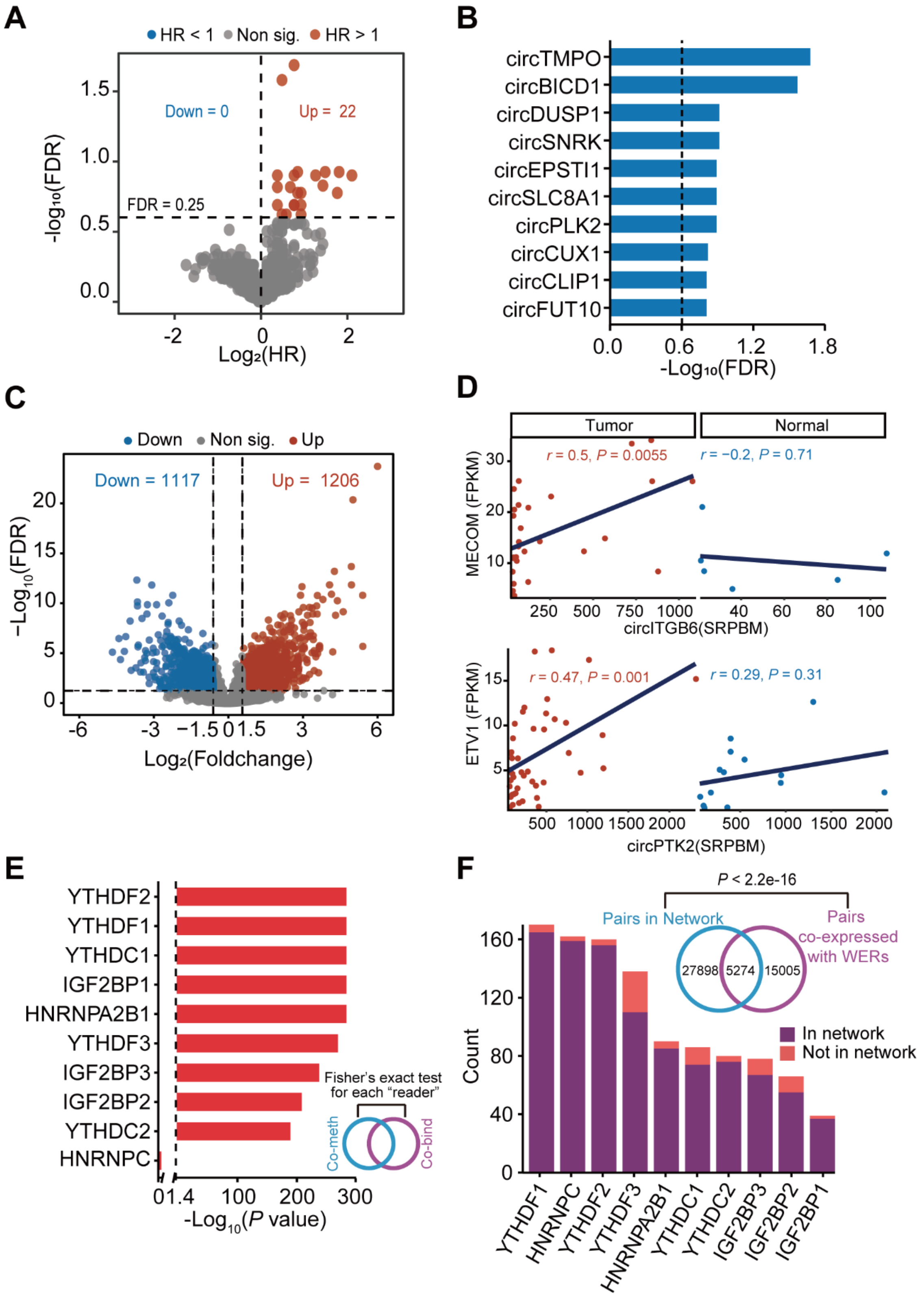

Fig. S4. Additional figures related to **Fig. 4**. (**A-B**) The correlation between OS and m^6A level of differentially methylated m^6A-circRNAs in PDAC. Volcano plot depicting $\log_2$ hazard ratios (HRs) and $-\log_{10}$ (FDR) values of differentially methylated m^6A-circRNAs in the Cox proportional hazards model of OS. The horizontal dashed line in (**A**) and vertical dashed line in (**B**) correspond to an FDR = 0.25. Top 10 m^6A-circRNAs significantly associated with OS were showed in B. (**C**) Volcano plot for differential expression analysis of mRNAs between PDAC tumor and normal tissues. The horizontal dashed line corresponds to an FDR = 0.05. The vertical dashed line corresponds to a fold change ≥ 1.5 (upregulation) and a fold change ≤ -1.5 (downregulation). (**D**) Additional examples for the gain of co-expression in PDAC tumor compared to normal tissue samples. (**E**) Top m^6A "readers" that tend to bind co-methylated pairs (both the circRNA and mRNA were m^6A-modified in the coexpression network). Co-bind infer that a "reader" bind to both circRNA and DEmRNA of a pair. The *P* value was calculated with Fisher's exact test. (F) Significant overlap between circRNA-mRNA pairs in the co-expression network and circRNA-mRNA pairs correlated with m^6A "readers". Counts of m^6A reader correlated mRNAs in the circRNA-mRNA co-expression network.

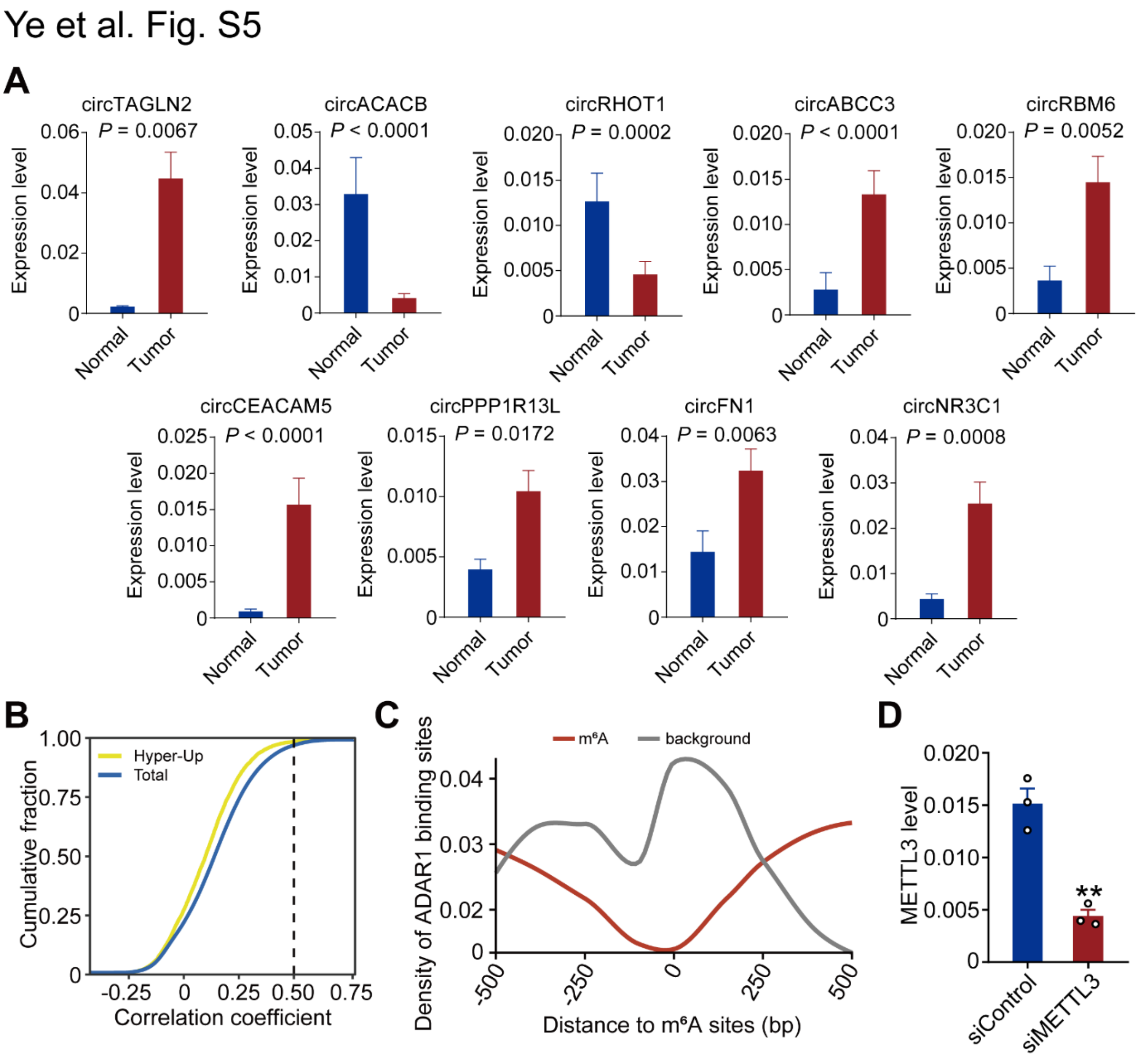

Fig. S5. Additional figures related to **Fig. 5**. (**A**) Validation of the differential expression of 9 circRNAs with qRT-PCR after RNase R treatment. (**B**) The plot of the cumulative density function (CDF) for the correlation coefficients between the expression levels of hyper-up circRNAs and "readers", and the correlation coefficients between all circRNAs was background. (**C**) The distribution of binding sites for ADAR1 in the flanking regions (500 nt) around the m^6A peak center was gradually decreased, compared with background (generated by the shuffleBed

function of the Bedtools). (**D**) METTL3 knockdown in PANC-1 cell was confirmed by qRT-PCR.

Ye et al. Fig. S6

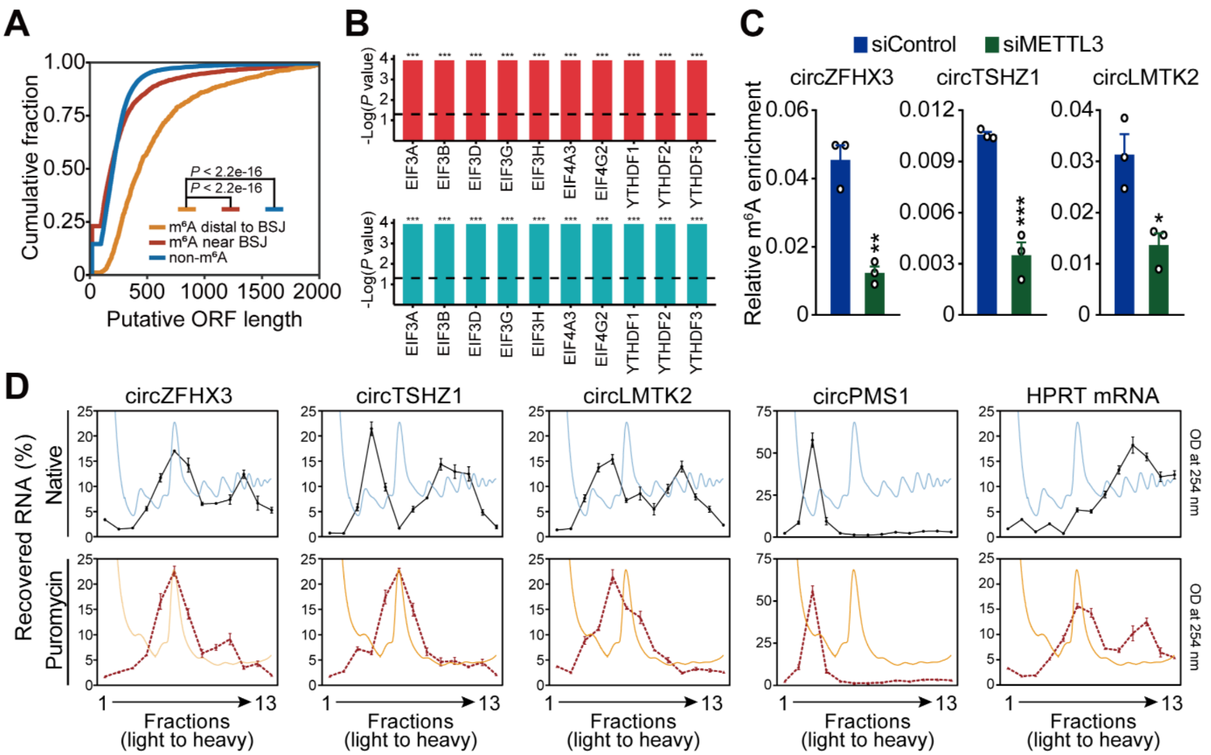

Fig. S6. Additional figures related to **Fig. 6**. (**A**) ORF length of BSJ-distal m^{6}A-circRNAs, BSJ-proximal m^{6}A-circRNAs and non-m^{6}A-circRNAs. (**B**) Bar plot showed the -log$_{10}$(*P* value) of permutation test for the preferential binding of Eukaryotic initiation factors (EIFs) and YTHDF1,2,3 to BSJ-distal m^{6}A-circRNAs over BSJ-proximal m^{6}A-circRNAs (upper panel; red bars) and BSJ-distal m^{6}A-circRNAs over non-m^{6}A-circRNAs (bottom panel; blue bars). n.s., $P > 0.05$; *, $P \leq 0.05$; **, $P < 0.01$; ***, $P < 0.001$. (**C**) Significant reduction of m^{6}A methylation on indicated circRNAs were observed after METTL3 knockdown in PANC-1 cells cells (n = 3). (**D**) Cytoplasmic extracts from PANC-1 cells, either untreated (native, upper) or treated with puromycin (under), were loaded on 15%–50% sucrose gradients. Absorbance at 253 nm was measured and fractions were collected. Fraction density decreases from left to right. Individual

fractions were analyzed by qRT-PCR and are represented as a percentage of total RNA in each fraction. CircPMS1 and HPRT mRNA represent negative and positive controls, respectively.

Table S1. Baseline demographic and clinical characteristics of individuals with PDAC in this Study.

Sample ID	Neural invasion	Vascular invasion	Lymph node metastasis	Tumor stage[1]	Tumor tissue	Normal tissue	Remarks
PDAC_1	No	No	No	II	Yes	NA	
PDAC_2	No	No	No	III	NA	Yes	
PDAC_3	Yes	Yes	Yes	II	NA	Yes	
PDAC_4	No	No	No	II	NA	Yes	
PDAC_5	Yes	No	Yes	II	Yes	NA	
PDAC_6	No	No	No	I	Yes	NA	
PDAC_7	Yes	No	Yes	II	NA	Yes	
PDAC_8	No	No	Yes	II	Yes	NA	
PDAC_9	No	No	No	III	Yes	NA	
PDAC_10	No	No	No	I	Yes	NA	
PDAC_11	No	No	No	I	Yes	Yes	Paired
PDAC_12	Yes	No	No	II	Yes	NA	
PDAC_13	Yes	No	Yes	II	Yes	NA	
PDAC_14	No	No	Yes	II	NA	Yes	
PDAC_15	No	No	No	II	Yes	NA	
PDAC_16	No	No	No	IV	Yes	NA	
PDAC_17	Yes	No	No	II	Yes	Yes	Paired
PDAC_18	Yes	Yes	No	II	Yes	Yes	Paired
PDAC_19	No	No	Yes	II	Yes	NA	
PDAC_20	Yes	Yes	No	II	Yes	NA	
PDAC_21	Yes	Yes	Yes	II	Yes	NA	
PDAC_22	No	No	Yes	II	Yes	NA	
PDAC_23	Yes	Yes	Yes	IV	Yes	NA	
PDAC_24	No	No	Yes	II	Yes	NA	
PDAC_25	No	Yes	Yes	II	Yes	Yes	Paired
PDAC_26	No	No	No	II	Yes	Yes	Paired
PDAC_27	Yes	No	No	II	Yes	NA	
PDAC_28	Yes	No	Yes	II	Yes	Yes	Paired
PDAC_29	No	No	Yes	II	Yes	NA	
PDAC_30	Yes	No	Yes	II	Yes	Yes	Paired
PDAC_31	No	No	Yes	II	Yes	Yes	Paired
PDAC_32	No	No	Yes	II	Yes	Yes	Paired
PDAC_33	Yes	No	Yes	II	Yes	Yes	Paired
PDAC_34	No	No	No	I	Yes	NA	
PDAC_35	Yes	No	Yes	II	Yes	NA	
PDAC_36	No	No	No	I	Yes	NA	
PDAC_37	Yes	Yes	No	II	Yes	NA	

PDAC_38	No	Yes	No	IV	Yes	Yes	Paired
PDAC_39	No	No	No	IV	Yes	Yes	Paired
PDAC_40	No	No	Yes	II	Yes	NA	
PDAC_41	Yes	Yes	Yes	II	Yes	Yes	Paired
PDAC_42	Yes	No	No	II	Yes	Yes	Paired
PDAC_43	No	No	No	III	Yes	NA	
PDAC_44	No	No	No	II	Yes	NA	
PDAC_45	No	No	Yes	II	Yes	Yes	Paired
PDAC_46	No	No	No	II	Yes	Yes	Paired
PDAC_47	Yes	No	Yes	II	Yes	NA	
PDAC_48	Yes	Yes	Yes	II	Yes	NA	
PDAC_49	No	No	No	II	Yes	NA	
PDAC_50	Yes	Yes	Yes	II	Yes	Yes	Paired
PDAC_51	No	No	Yes	II	Yes	NA	
PDAC_52	Yes	No	Yes	IV	Yes	NA	
PDAC_53	No	No	No	III	Yes	NA	
PDAC_54	No	No	No	IV	Yes	NA	
PDAC_55	No	No	No	I	Yes	Yes	Paired
PDAC_56	No	Yes	No	II	Yes	NA	
PDAC_57	No	No	No	IV	Yes	NA	
PDAC_58	No	No	No	IV	Yes	NA	

[1]Tumor TNM staging were reviewed by at least 3 pathologists and defined according to the American Joint Committee on Cancer (AJCC) 7th edition.

Table S2. The primers sequences used in this study.

**Primers for circRNA MeRIP-qPCR and quantitative real time-PCR**		
Gene symbol	Forward primer (5'→3')	Reverse primer (5'→3')
circTAGLN2	TGAGAACACTCCCTGTCCCAA	CCATATGCAGGTCCCCTGTT
circACACB	TCCTCCCAGCGGATTCACTA	AACATGGTCAGAGACTGCCG
circRHOT1	CTACTTGGACTGTGCTTCGAC	AGTGCAATCAGGAGGTATTTTCAG C
circABCC3	ATTCCACTCAACGGAGCTGTG	GCGCGAGTCCTTCAATTTCAT
circCEACAM5	AAGAAATGACGCAAGAGCCTATG	CCCGAAAGGTAAGACGAGTCTG
circPPP1R13L	GAAAGCCTGGAACGAGTCTGA	GCGCTAGTGAGGTTGTCCT
circFN1	GAGAATAAGCTGTACCATCGCAA	CGACCACATAGGAAGTCCCAG
circCPB1	ACCAAGTACACATATGGCCCG	CAGGACGTAGCTGGCAACATA
circRBM6	GGGAAGGGCCAACTTTCCG	TGGAACGATAGATACGCTTTAGC
circNR3C1	ATAGCTCTGTTCCAGACTCAACT	ATCCAGGTGTAAGTTCCTGAAACC T
circFCHO2	TGTATTGTGAAGATCACTGGTGATA	TAACTTGGTGGGATCTGCTCCTT
circPOSTN	GTCTCCTGTCTGATTTTAGCCA	AATAAATGACCATCACCACCTT
circVTI1A	ACATCTGCTCGATAACACAGA	TCTCTGAGGAATTCCCATCAT
circPEAK1	ATGCATTACTACAGGACTCAG	CTATGTGTTACAGCAGCTCT
circPTPRH	ACATCACCGTGGATAGACTTG	GAGATGGAGCTGGTGGTCTGA
circZFHX3	CAGTTCATGATGAGCGGATT	GTCACAGCCTTCCATGGTAA
circTSHZ1	ACTGCACCTTAGTAAGACCCA	CTCCTCGTGCTCTTCATCTAT
circ-LMTK2	AGAAGGAAAAGAAGGCAGTCA	ACTTGGTTGAGGACATCTAAGT
β-Actin	CAGGGCGTGATGGTGGGCATG	GTAGAAGGTGTGGTGCCAGATT
**Primers for mRNA quantitative real time-PCR**		
TAGLN2	GAACTTCTCGGATAACCAGCTGCAA	AGTTCTGGAAGTTCTCGCGTCCA
ACACB	TAATTCAGCAGGCAGGACAGGTGT	ACGATCTCTTCGTAAGCCAGA
RHOT1	ATAGAATTTAGCTTGGCCATTAGT	ATTAAATTAACCTACACCTTCAT
ABCC3	ATCCAGAACTGCACTCTTCAGGAA	TGCGCAGCAGCTGGAGCTCA
CEACAM5	TATTACCGTCCAGGGGTGAACCTCA	ACAGGTGAAGGCCACAGCATCCTT
PPP1R13L	ATGAAACACATGGATCTGAAGCAGA	TCTGGAATGCCTCGCTGTCCAT
FN1	TAAGTGAGGCTCACATGGACTTT	ATGGGGCTTGTTGTCACTTACCTTC
CPB1	TACTGATAAGCAACCTGAGAAATGT	TAAACTTACTTGTATTGTAGTTC
RBM6	AGTCAAGGAAAGTCAAGTAGCAAG	ACCTGTTACAGCCTACCTGATAGT
NR3C1	ACTCTGAACTTCCCTGGTCGAACAG	TTTATAGAAGTCCATCACATCTC
β-Actin	CAGGGCGTGATGGTGGGCATG	GTAGAAGGTGTGGTGCCAGATT
**Primers for nascent pre-mRNA detection**		
pre-POSTN	ATGGAATCAAAATGGTGAACAA	AAAAGTTGCTTACCAGAATCAGGA A
pre-VTI1A	GATCACCAGCAAGATTGCGAGGGTC	CACTTACTCGGGCGGCGTTCCA
pre-PEAK1	TCAGGCTAACCAGTGACAAACC	ATGAATCCATCCCAGTAACAAT
pre-PTPRH	GTAGCCAGTTCCACGCAGAGCC	TGAGCTAAGCCCAGTTCACCACT

pre-TAGLN2	CCAATTAAACACCCTAGCCAAGAAC	CTGGAGTTACAATAGGCGGGAAA
pre-CEACAM5	TTCTACACCCTACACGTCATAAAGTC	ACTAAATGCCCAAACCCTAACA
pre-RBM6	GAGTCACGCTTAGGACATCAAA	TGGTATGTGCCCTGACAAACTA

Table S3. Top 14 features for the construction of random forest model.

Features	Description	Additional information
Exon count	The count of exons per circRNA	
Gene feature	The genes feature that circRNA located in	5'UTR,CDS,3'UTR,exon,intron
CircRNA SRPBM[1]	The expression level of circRNAs, normalized by SRPBM	
Linear m^6A peak	Whether linear counterpart RNAs[2] has a m^6A peak within circRNA locus.	Yes or Not
Linear peak number	The m^6A peak count of linear counterpart RNAs within circRNA locus	If Linear m^6A peak = “Not” , the feature value is 0
Free energy	The free energy of each circRNA	The free energy is calculated by RNAfold (v2.4.13) package (parameter: --circ)
Length	The length of each circRNA	
GC content	The percentage of guanine (G) or cytosine (C) in circRNAs.	
Max exon length	Length of the longest exon in circRNAs	
Exon length average	Average length of exons in circRNA	
RRACH motif[3]	Whether circRNA contain RRACH motif	Yes or Not
TE[4] count in 5’ splicing site (ss)	The number of Transposable elements in upstream 2000 bp of 5’ss	
TE count in 3’ splicing site (ss)	The number of Transposable elements in downstream 2000 bp of 3’ss	
House-keeping genes	Whether host gene of circRNA is a house-keeping gene .	Yes or Not

[1]SRPBM, spliced reads per billion mapped reads, a normalization method for read count of circRNAs;

[2] linear counterpart RNAs: linear isoforms of host gene that also generate circRNA;

[3]RRACH motif: “RRACH” (R = G or A; H = A, C or U), the consensus motif of m^6A modification;

[4]TE, Transposable elements.

Table S4. The performance of Circm6A in the identification of circRNAs on the simulated datasets.

**Method**	**RNA-seq datasets[1]**				
	**Detected circRNAs**	**TP[2]**	**S(%)[3]**	**P(%)[4]**	**F1 score[5]**
**Circm6A**	**18554**	**18520**	**98.70**	**99.82**	**0.99**
ACFS	8445	8448	45.01	99.96	0.62
AutoCirc	18265	16564	88.28	90.69	0.89
CIRCexplorer2	18038	18036	96.13	99.99	0.98
circRNA_finder	17488	17470	93.11	99.90	0.96
CIRI2	18484	18441	98.28	99.77	0.99
DCC	18142	18132	96.64	99.94	0.98
Find Circ	16741	16592	88.43	99.11	0.93
MapSplice	16720	16719	89.11	99.99	0.94

[1]RNA-seq datasets were generated by in-house simulator to evaluate the performance of each method in detecting circRNAs.

[2]TP: True positive ratio;

[3]S: Sensitivity;

[4]P: Precision;

[5]F1 score = (2 × precision × sensitivity)/ (precision + sensitivity).

Table S5. Compare the performance of Circm6A in the identification of m^6A-circRNAs with other tools using simulated data

Method	MERIP-seq datasets[1]				
	Detected circRNAs	TP[2]	S(%)[3]	P(%)[4]	F1 score[5]
**Circm6A(high)[6]**	**1522**	**1511**	**98.82**	**99.28**	**0.99**
**Circm6A(total)[7]**	**1885**	**1512**	**98.89**	**80.21**	**0.89**
ACFS	905	757	49.51	83.65	0.62
AutoCirc	2870	1503	98.30	52.37	0.68
CIRCexplorer2	3005	1527	99.87	50.82	0.67
circRNA_finder	2873	1484	97.06	51.65	0.67
CIRI2	1933	1522	99.54	78.74	0.88
DCC	1865	1529	100.00	81.98	0.90
Find Circ	2678	1481	96.86	55.30	0.70
MapSplice	1644	1486	97.19	90.39	0.94

[1]MERIP-seq datasets were generated by in-house simulator to evaluate the performance of each method in detecting m^6A-circRNAs.

[2]TP: True positive ratio;

[3]S: Sensitivity;

[4]P: Precision;

[5]F1 score = (2 × precision × sensitivity)/ (precision + sensitivity)

[6]Circm6A(high): high confidence m^6A-circRNAs of Circm6A.

[7]Circm6A(total): high and low confidence m^6A-circRNAs of Circm6A.
