# Supplementary material for: Genome-wide identification and characterization of circular RNA m6A modification in pancreatic cancer
Source: Genome Med. 2021 Nov 19;13:183. doi: 10.1186/s13073-021-01002-w (PMC8605608; doi:10.1186/s13073-021-01002-w)
Supplement: Supplementary file 4 — Additional file 4. uncropped blot for Fig. S3A and Fig. 3B. [file 13073_2021_1002_MOESM4_ESM.pdf]

Uncropped blot for Fig. S3A

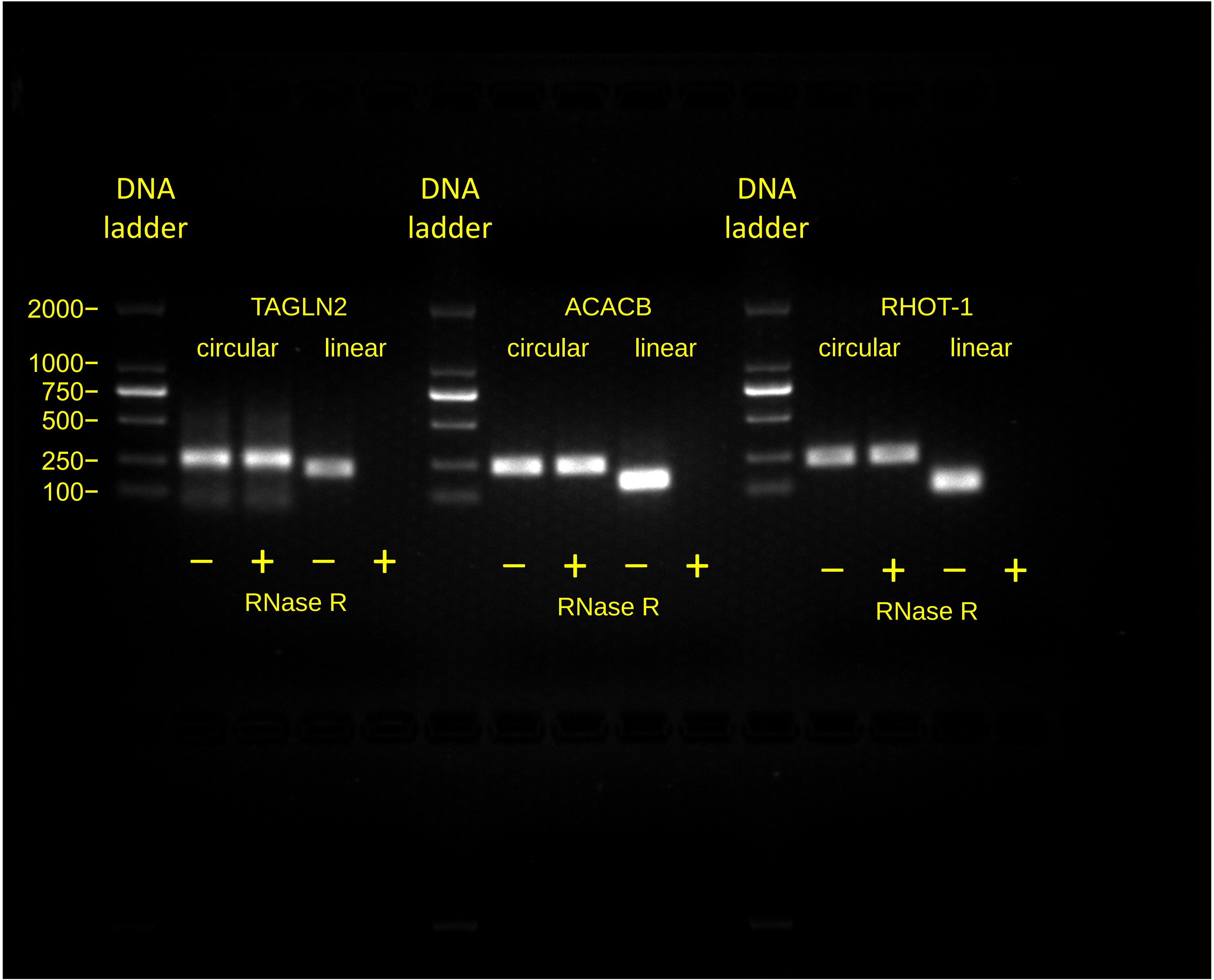
DNA ladder
DNA ladder
DNA ladder
2000
1000
750
500
250
100
TAGLN2
circular
linear
ACACB
circular
linear
RHOT-1
circular
linear
− + − +
RNase R
− + − +
RNase R
− + − +
RNase R

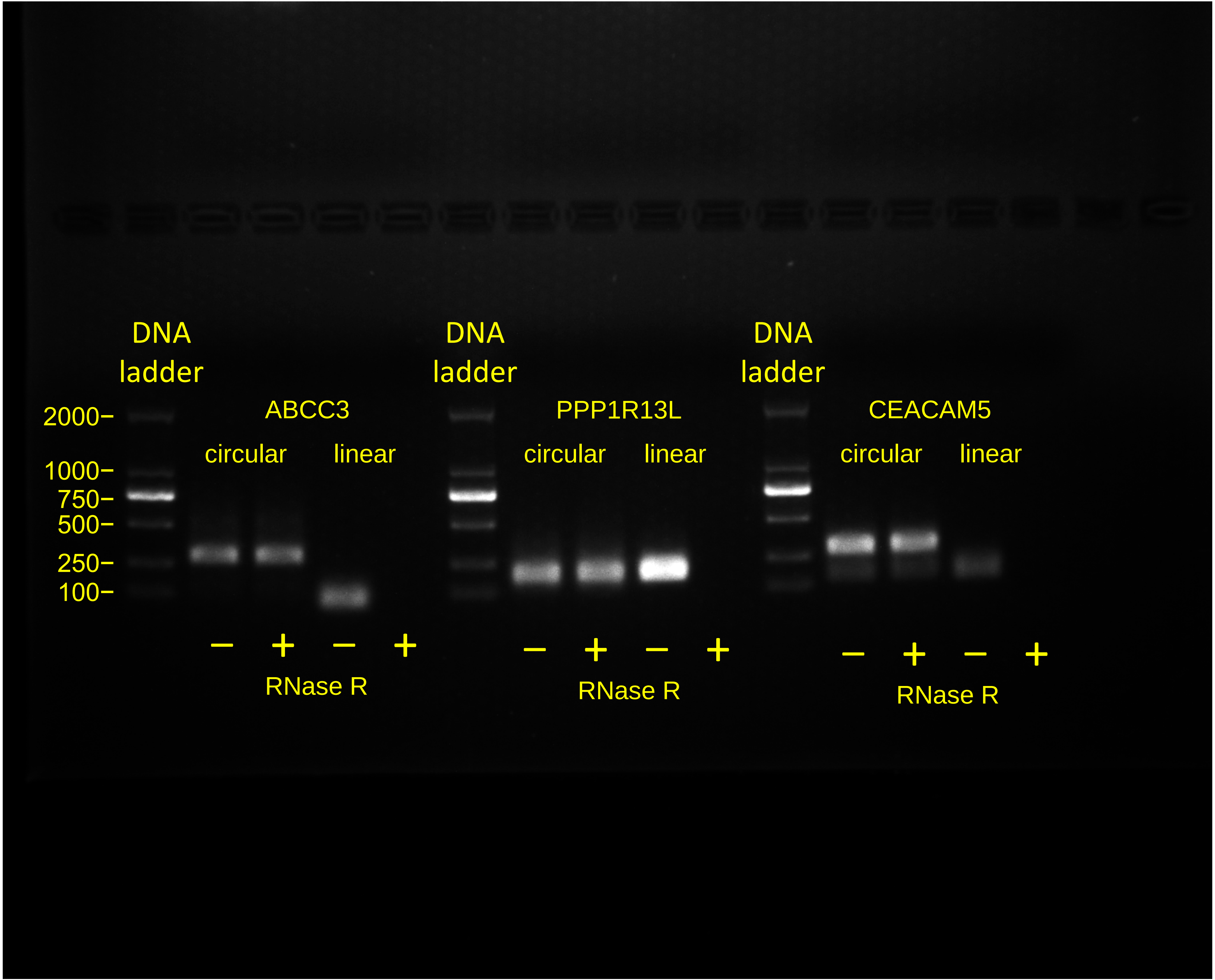
DNA ladder
DNA ladder
DNA ladder
2000
1000
750
500
250
100
ABCC3
PPP1R13L
CEACAM5
circular
linear
circular
linear
circular
linear
− + − +
− + − +
− + − +
RNase R
RNase R
RNase R

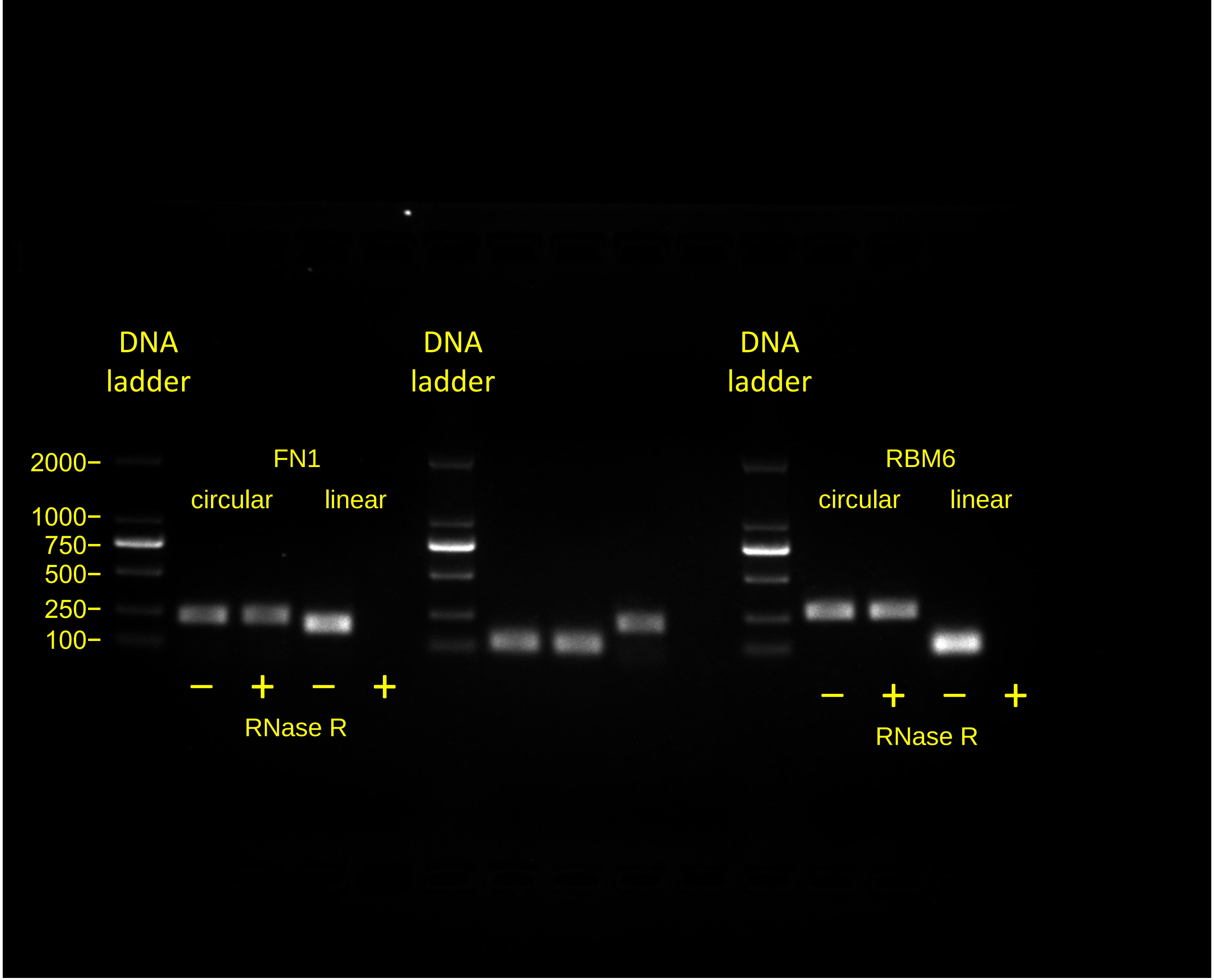
DNA ladder
DNA ladder
DNA ladder
2000–
1000–
750–
500–
250–
100–
FN1
circular
linear
RBM6
circular
linear
– + – +
RNase R
– + – +
RNase R

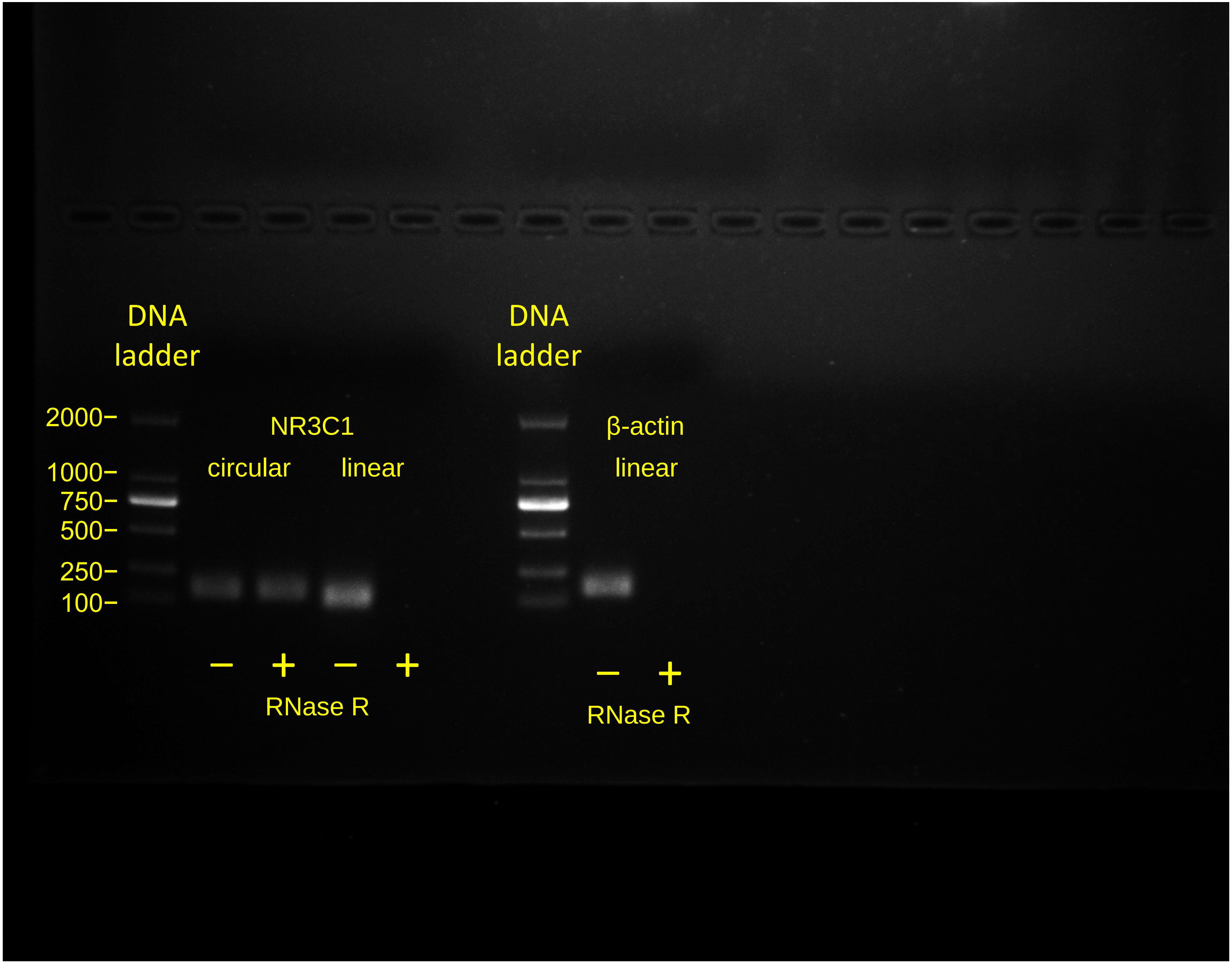
DNA ladder
2000–
1000–
750–
500–
250–
100–
NR3C1
circular
linear
– + – +
RNase R
DNA ladder
β-actin
linear
– +
RNase R

Uncropped blot for Fig. 3B

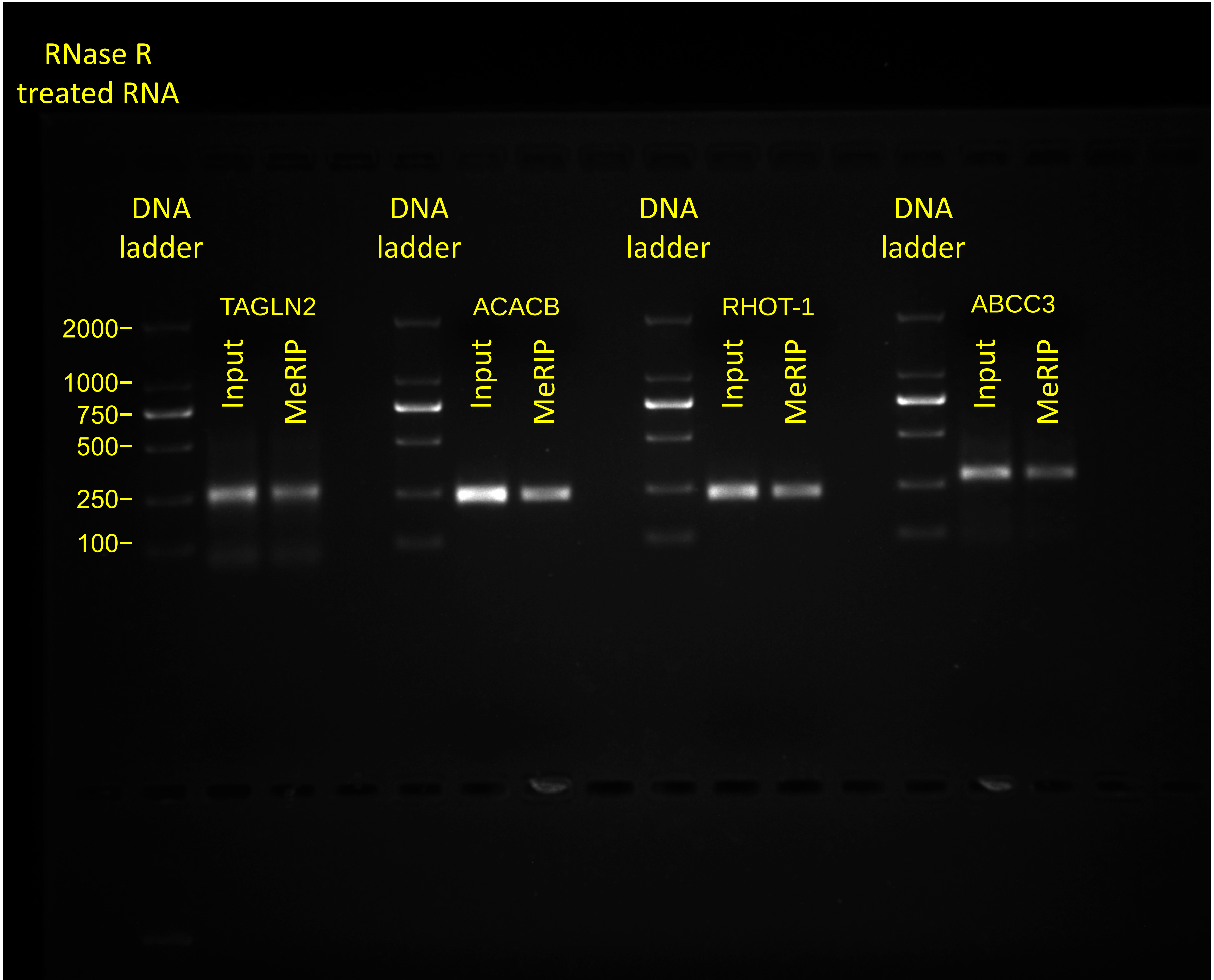
RNase R
treated RNA
DNA ladder
DNA ladder
DNA ladder
DNA ladder
TAGLN2
ACACB
RHOT-1
ABCC3
2000
1000
750
500
250
100
Input
MeRIP
Input
MeRIP
Input
MeRIP
Input
MeRIP

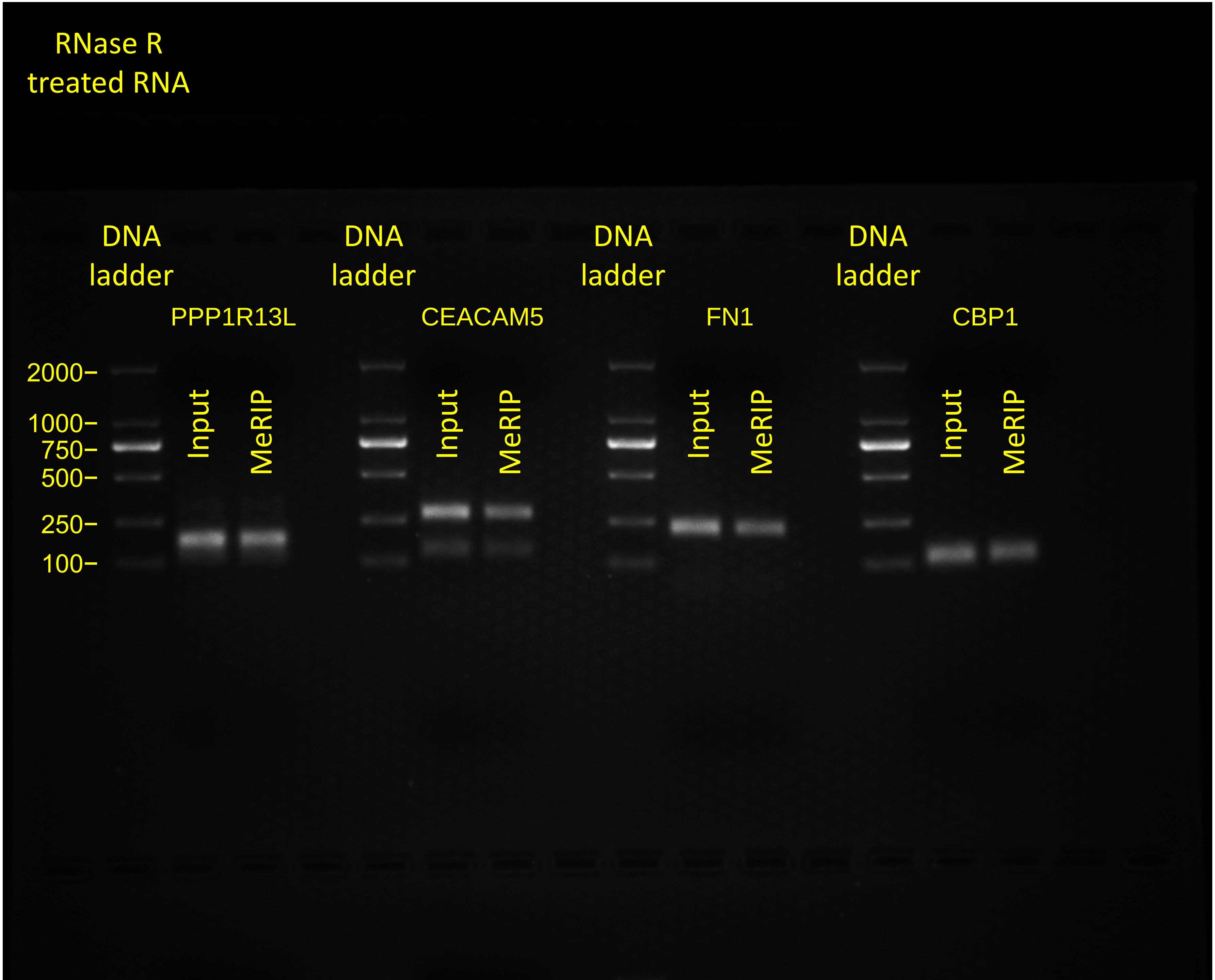
RNase R
treated RNA
DNA ladder
DNA ladder
DNA ladder
DNA ladder
PPP1R13L
CEACAM5
FN1
CBP1
2000–
1000–
750–
500–
250–
100–
Input
MeRIP
Input
MeRIP
Input
MeRIP
Input
MeRIP

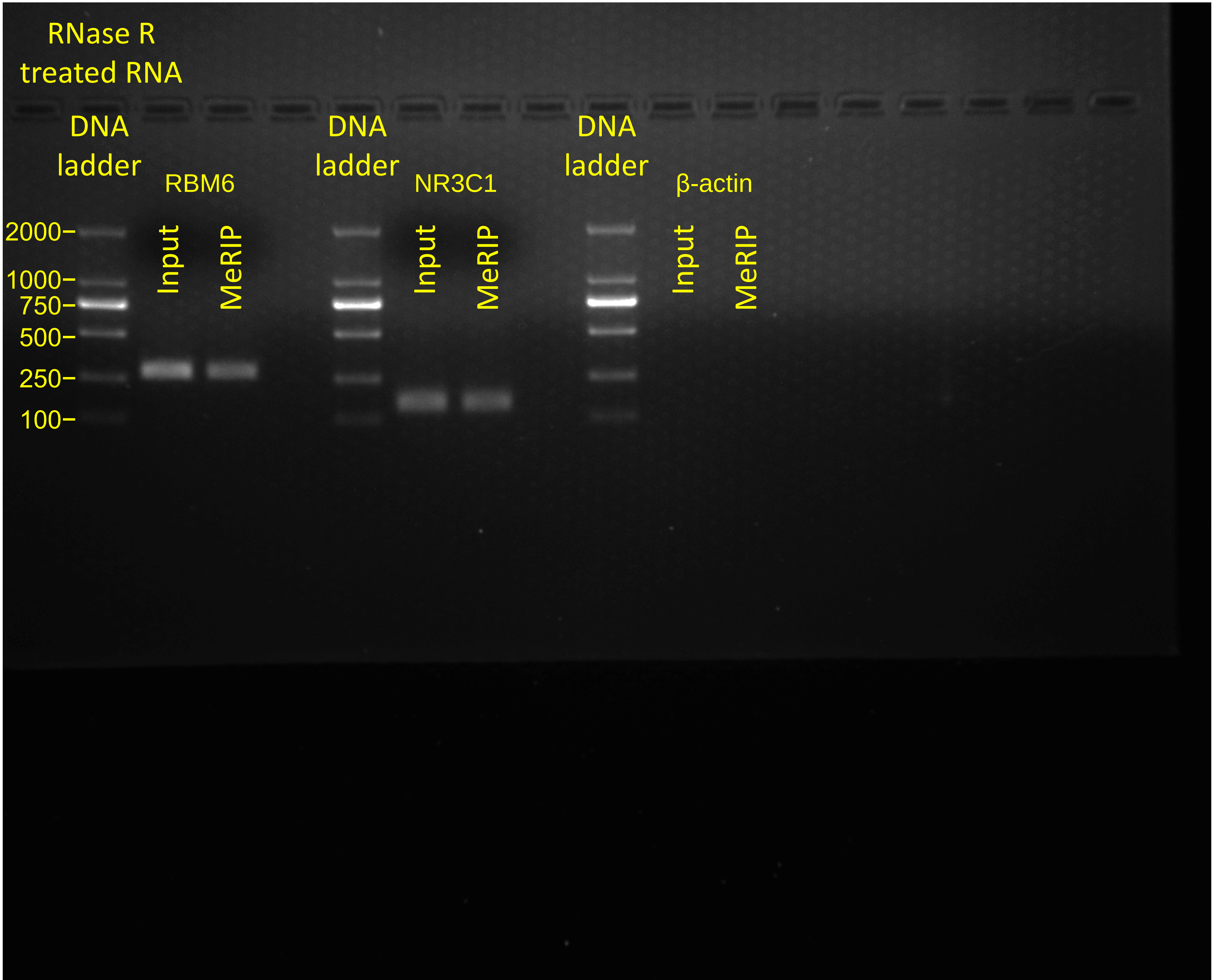
RNase R
treated RNA
DNA
ladder
RBM6
Input
MeRIP
DNA
ladder
NR3C1
Input
MeRIP
DNA
ladder
β-actin
Input
MeRIP
2000
1000
750
500
250
100

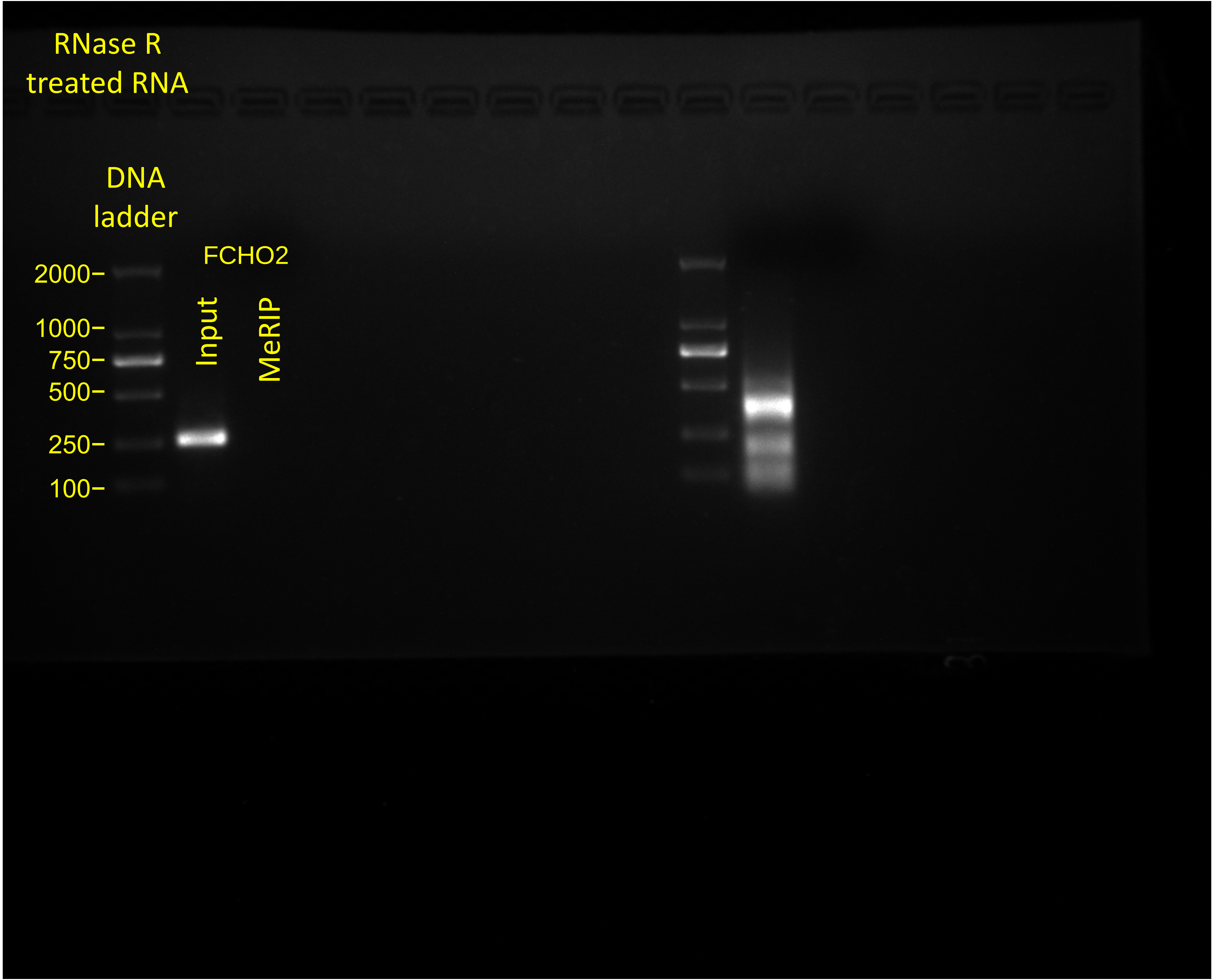
RNase R
treated RNA
DNA
ladder
FCHO2
Input
MeRIP
2000
1000
750
500
250
100
